# Exploring Technological Solutions for Interoperability Between Patient Electronic Medical Records and Clinical Registries: Scoping Review

**DOI:** 10.2196/82380

**Published:** 2026-05-25

**Authors:** Erika Haynes, James Brannigan, Jessica Suna, Reid Malseed, Alana Delaforce, Rachel Mulvenney-Fenner, Katherine Alog-Daroya, Karin Plummer, Craig McBride, Roy Kimble, Bronwyn Griffin

**Affiliations:** 1 School of Nursing and Midwifery, Griffith Health, Griffith University, 170 Kessels Road, Nathan, Brisbane, Queensland, 4111, Australia, 61 3735 7111; 2 Children's Health Queensland, Hospital and Health Service, Brisbane, Australia; 3 The University of Queensland, Brisbane, Australia; 4 Central Queensland University, Rockhampton, Australia; 5 CSIRO, Brisbane, Australia

**Keywords:** registry, electronic medical record, interoperability, data extraction, data transfer

## Abstract

**Background:**

The use of electronic medical records (EMRs) and clinical registries has transformed health care delivery by improving data management, care coordination, and research capacity. However, the full potential of these technologies can only be realized through effective interoperability, thereby reducing the burden of manual data entry and enhancing the use of real-world clinical data.

**Objective:**

This review examines technologies that enable automated data extraction and transfer, which promote interoperability between EMRs and clinical registries.

**Methods:**

A search of PubMed, CINAHL, Embase, and Web of Science, including studies published between January 2013 and April 2025, was registered with Open Science Framework a priori and involved three key concepts: (1) “registry,” (2) “electronic medical records,” and (3) “interoperability.” A 2-phase screen identified studies evaluating technologies that facilitate automated data extraction or interoperability. Automation was defined as fully automated, where data are extracted and transferred without human intervention, or semiautomated, where extraction or transfer is predominantly automated but may include manual validation. Only technologies supporting ongoing database integration were eligible for inclusion. Screening, data extraction, and synthesis were conducted by multiple independent reviewers. Technology experts provided extensive input and guidance throughout to ensure the accuracy and relevance of the extracted information.

**Results:**

Overall, 36 studies met the inclusion criteria, representing 12 countries across 5 continents and addressing a wide range of acute and chronic health conditions. Epic was the most frequently reported EMR system, while the most common registry platforms were REDCap (Research Electronic Data Capture; Vanderbilt University), structured query language (SQL) server database, and EMR-embedded solutions. Most approaches centered around extracting data from structured formats (n=18), or a combination of both structured and unstructured formats (n=10), emphasizing the central role of structured EMR data in current automated extraction approaches.

**Conclusions:**

This review advances understanding of interoperability between EMRs and clinical registries by uniquely examining automated and sustainable solutions for data exchange, extending beyond prior work that has largely focused on technologies designed for isolated systems or study-specific data extraction. A novel contribution of this review is the synthesis of context-specific considerations derived from reported implementations, providing a comprehensive overview of how technology selection and implementation are shaped by the context in which they are deployed. While these advancements have reduced reliance on inefficient, error-prone, and resource-intensive manual processes, ongoing challenges in data standardization, seamless integration, and long-term sustainability are compounded by poor and inconsistent reporting across studies. Future efforts should follow comprehensive reporting guidelines, adhere to robust governance principles, and incorporate implementation science frameworks, to not only enable meaningful comparison and synthesis in future research, but also to ensure that technologies can be effectively, feasibly, and sustainably integrated within health care contexts, while upholding the ethical and equitable use of health care data.

## Introduction

In health care, the integration of innovative technology has driven unprecedented progress in communication and information systems, resulting in significant impacts on the efficiency, accuracy, and quality of health care delivery [1]. Electronic medical records (EMRs) and clinical registries exemplify the sophisticated application of technology to streamline data management, enhance patient care coordination, and support robust clinical research [2]. Clinical registries in particular are increasingly leveraged for research, quality assurance, and benchmarking, allowing health care providers and researchers the ability to maximize resources and improve patient outcomes in an era of growing demands and limited funding [3,4]. However, the full potential of these systems can only be realized through effective interoperability, which ensures seamless data exchange and integration across diverse health care platforms [5].

Interoperability is broadly defined as “the ability of two or more systems to work together, regardless of different interfaces, platforms, and technologies adopted” in a way that facilitates data-sharing and data-use in improving health care delivery [6]. In this context, the promise of interoperability between patient EMRs and clinical registries represents enormous potential to minimize the burden of data input, management, and maintenance, while maximizing research productivity in line with real-world experiences [7-9]. The issue therein not only stems from technical limitations but also encompasses concerns regarding maintaining information fidelity alongside data standardization, ensuring data privacy and security, and navigating regulatory constraints [9,10]. Overcoming these challenges is pivotal to harnessing the full potential of registries and EMRs, moving toward a more integrated and efficient health care technology ecosystem.

In contemporary health care, EMRs play a crucial role in capturing comprehensive patient data across various clinical encounters [11]. The data, documented in either a structured format (eg, predefined fields such as diagnosis codes, lab results, and medication lists) or an unstructured format (eg, free-text clinical notes, summaries, and narrative reports) [12], facilitates patient care by centralizing all patient information in one location. This centralization keeps multidisciplinary team members informed, supports clinical decision-making, and ensures continuity of care across diverse health care settings [13,14]. Typically, these same data elements are also leveraged in clinical registries to systematically collect, study, and interpret patient data relevant to specific medical conditions [15,16], thus filling broad knowledge and evidence gaps that are challenging and costly to capture using traditional research methods [15,17]. At present, the population of registry data predominantly relies on manual methods, where data are extracted from EMRs and transcribed into the correct format for registry use [18-20]. Despite the translational benefits of clinical registry data use, this current process is time-consuming, error-prone, resource-intensive, and often constrained by the availability of ongoing funding [20-24].

Automated data extraction and transfer technologies have emerged as critical tools to improve interoperability, reduce data entry burden, and enhance the timeliness and completeness of health information for research, quality improvement, and policy development [25,26]. However, while these technological advancements offer significant benefits, there is currently no universally accepted “gold standard” methodology, and challenges surrounding adoption are not yet fully understood. Successful integration of these tools requires not only technical accuracy in extracting and transmitting standardized data, but also robust measures to protect patient privacy, ensure data security, and comply with regulatory requirements [25,27,28]. Adoption is further shaped by organizational, technical, and cultural barriers, underscoring the value of implementation science frameworks to guide effective and sustainable integration [29,30]. Within this context, this review primarily aims to explore existing automated data extraction and transfer processes from EMRs to clinical registries, with a focus on technologies used, data fidelity, and governance.

The specific review questions are:

1. What technologies are being used to facilitate interoperability through automated data extraction and transfer from EMRs to clinical registries or databases?
2. To what degree has the implementation of automated data extraction and transfer tools successfully enabled accurate and complete transmission of data in a standardized format?
3. What measures have been implemented to safeguard data privacy, ensure security, and comply with regulatory requirements during automated data extraction and transfer?
4. What implementation challenges have been reported in the adoption of these technologies, and what implementation frameworks have been used to support their effective integration into clinical data systems?

## Methods

### Study Design

The PRISMA-ScR (Preferred Reporting Items of Systematic Reviews and Meta-Analyses extension for Scoping Reviews) was used to conduct this review [31,32]. The review protocol was registered with Open Science Framework prior to study commencement [33].

### Search Strategy

The search strategy was developed in consultation with a university health librarian to find published studies relevant to the review questions. The search strategy comprised three key concepts: (1) registry, (2) electronic health records (EHR), and (3) interoperability (Table S1 in Multimedia Appendix 1). While both terms, EMR and EHR, were included in the search strategy to ensure comprehensiveness, this study uses EMR to collectively refer to both terms, except where the original technology name explicitly uses EHR.

The Systematic Review Accelerator Polyglot Search Translator automation tool [34] was used to convert this search strategy into the appropriate formats for 4 databases, including PubMed, CINAHL, Embase, and Web of Science. The reference lists of all included sources were hand-searched. Non-indexed publications were considered if identified through reference list hand-searching, but were not systematically sought beyond the selected databases. Gray literature was neither searched nor eligible, as the review focused exclusively on peer-reviewed studies. The search included all studies published between January 1, 2013, and April 30, 2025, inclusive.

### Eligibility Criteria

The core concept of interest was any study exploring technology enabling interoperability between EMRs and research databases, such as clinical registries. Automation was defined as either fully automated, in which data are extracted and transferred without human intervention, or semiautomated, in which extraction or transfer is predominantly automated but may include manual validation steps. This ability to automate the process of extracting and transferring data for research purposes was key for inclusion and, as such, any study using a manual extraction or transfer process was excluded. Publications focusing on technology to extract data for a single study, rather than for integration into ongoing databases or registries, were also excluded. Eligibility within this scoping review was not limited by a specific type of participant, population, or health care context. Similarly, the study design and the specific EMR or registry used did not affect inclusion. Only full-text, peer-reviewed, primary research studies available in English were considered for inclusion, ensuring sufficient detail on the relevant technology was available.

### Study Selection

Studies were uploaded into Covidence (Covidence systematic review software) for screening and de-duplication [35]. Four independent screeners (ED, JB, RM, and KA) completed a title and abstract screen. Conflicts were resolved by discussion between 2 reviewers (ED and JB). Once all conflicts were resolved, the same screeners independently completed the full-text screen, recording the rationale for study exclusion to ensure transparency.

### Data Extraction, Synthesis, and Quality Appraisal

Data were entered into Microsoft Excel using a data extraction tool developed a priori [36]. The data extracted are presented in Table S1 in Multimedia Appendix 2.

Each paper was extracted by at least 2 reviewers (ED, JB, RM, and KA), with 2 additional technology experts (JS and RM) contributing to the extraction process and providing technological guidance. Quality appraisal of individual sources was not conducted, as inclusion was based solely on relevance to the review aims, regardless of methodological rigor or risk of bias. This approach aligns with PRISMA-ScR guidance and reflects the broader intent of scoping reviews to provide a comprehensive overview of existing literature while mapping the breadth and nature of available evidence [32].

## Results

### Overview

**Figure 1.**
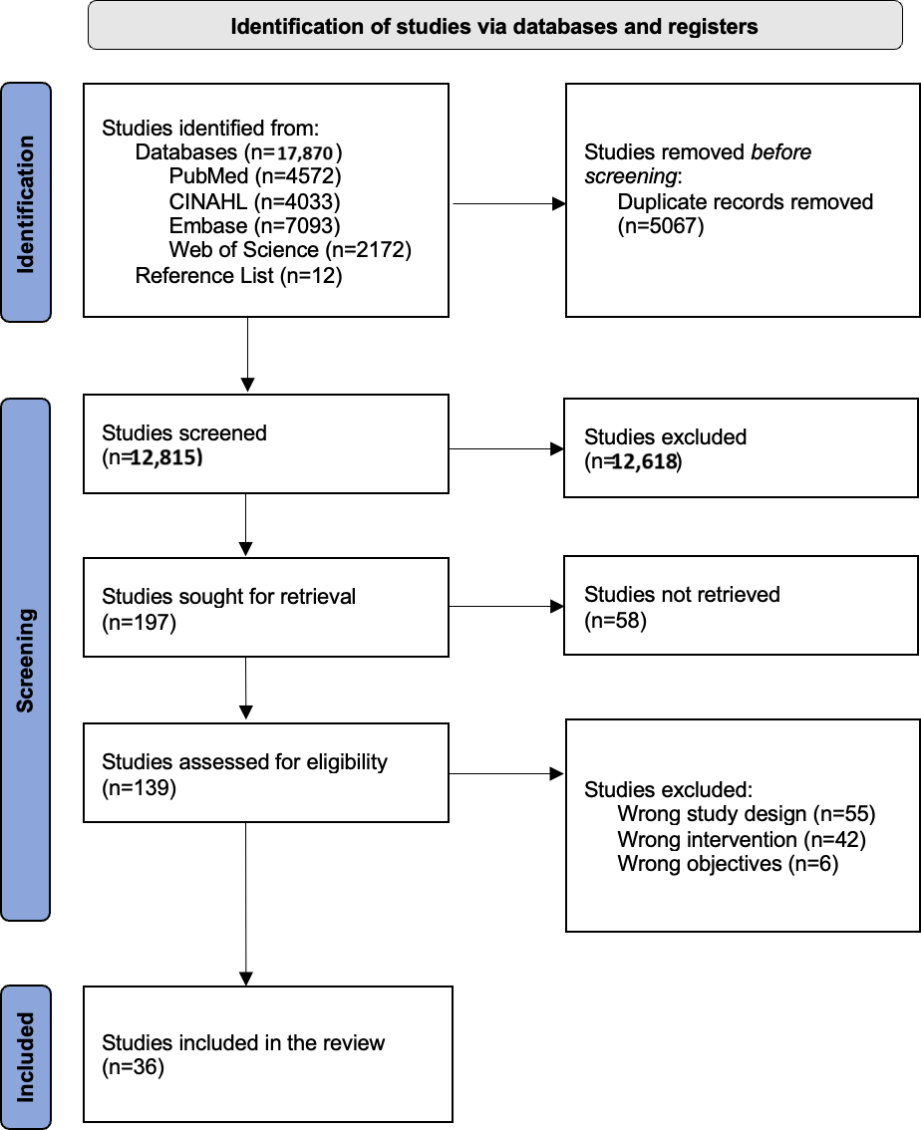
PRISMA (Preferred Reporting Items for Systematic Reviews and Meta-Analyses) flow diagram illustrating the identification, screening, eligibility assessment, and inclusion of studies published between January 2013 and April 2025 inclusive.

The search yielded 12,815 studies for screening. Overall, 36 studies [14,18-24,37-64] met the inclusion criteria and were included in the final review. A summary of the PRISMA (Preferred Reporting Items for Systematic Reviews and Meta-Analyses) flow chart is displayed in Figure 1.

### Study Characteristics

A total of 36 heterogenous studies [14,18-24,37-64] were included in the review (Table S1 in Multimedia Appendix 3). Twelve countries from 5 continents were represented, with the majority of studies originating from the United States. Twenty studies [14,18,19,24,37-52] were single-site. The studies addressed a wide range of acute and chronic health conditions, though 3 studies [41,53,54] did not specify the registry’s clinical focus. Patient data were extracted from between 50 and 1.4 million patients per study, with 16 studies [22,24,39,41,45,49-54,57-60,64] not clearly reporting the number of unique patients included. The most predominant health care setting type was outpatient care (n=7) [14,18,19,21,22,55,61], followed by a combination of settings (n=6) [52-54,57,62,63], inpatient care (n=4) [37,38,45,56], surgical (n=3) [47,49,59], and telehealth (n=1) [39]; 15 studies [20,23,24,40-44,46,48,50,51,58,60,64] did not clearly describe the setting type. The predominant study design was descriptive technical reports (n=20) [18-23,37,40,41,45,48,53-56,58,60,62-64], which detailed the development or integration of automated extraction technologies with EMR and registry systems, and pilot validation studies (n=13) [38,39,42-44,46,47,49-52,56,59], which evaluated data extraction systems on a smaller scale to assess accuracy or feasibility.

A variety of EMRs were used, with Epic (Epic Systems Corporation) being the most frequently used system, appearing in 12 studies [18,20,21,23,41,42,45,46,52,53,55,56]. Eight studies [39,40,43,44,50,57-59] did not specify the system used, with an additional 4 studies [60-63] reporting that multiple EMR solutions were used without specifying which systems. Similarly, 12 studies [18,24,38-41,43,57-59,62,63] did not specify the registry or database systems used. The most commonly used database systems included REDCap, structured query language (SQL) server database, and EMR-embedded solutions.

### Technological Approaches to Data Extraction and Transfer

**Figure 2.**
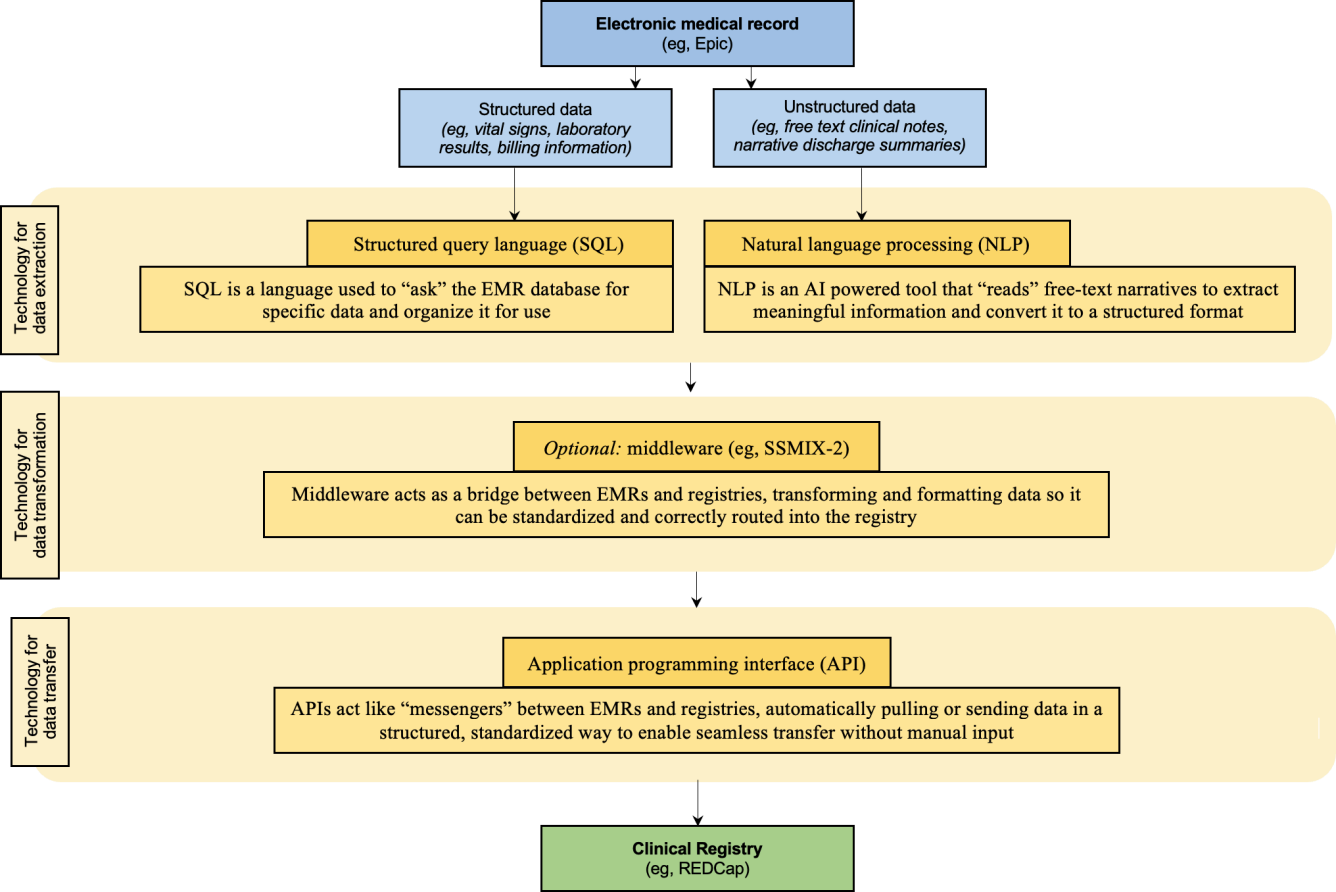
Common technological approaches supporting electronic medical record-to-registry data extraction and transfer. EMR: electronic medical record; REDCap: Research Electronic Data Capture; SSMIX-2: Standardized Structured Medical Information exchange version 2.

Various technologies were used to facilitate the automated extraction and transfer of data from EMRs onto clinical registries (Table S1 in Multimedia Appendix 4), with approaches often tailored to align with data formats and institutional requirements. Figure 2 provides a schematic overview of the main types of technological solutions identified in this review, and how they interface with EMRs and clinical registries. A total of 18 studies [14,19-21,23,24,45-48,53,55-58,60,61,63] extracted data from structured formats, 10 studies [18,22,37,40-42,50,52,62,64] extracted data from mixed formats (structured and unstructured), 6 studies [38,39,43,44,51,59] extracted data from unstructured formats, and the remaining 2 studies [49,54] did not report the format of the data source.

Data extraction from structured fields was most commonly performed using SQL querying methods, as reported explicitly in Abu-Rish Blakeney et al [37] (SQL database extraction, near real-time transfer), Bodagh et al [14] (SQL queries via Cerner Health Information Exchange and National Health Service Data Transfer Services, near real-time), and Kannan et al [21] (custom SQL-based extract, transform, load [ETL] pipeline, weekly extraction). Similar SQL-based approaches were inferred to have been used by Kariuki et al [19] and Nasir et al [55], based on their description of SQL-driven ETL pipelines, although detailed technical implementation was not fully described.

SQL enables automated extraction of clinical data from EMR databases by querying structured fields such as diagnosis codes, laboratory results, and medication lists. These data elements are typically stored in relational tables within the EMR. SQL scripts are written to select specific fields based on predefined criteria (eg, diagnosis and date range) and export them in a standardized format for registry ingestion.

Queries can be scheduled to run at regular intervals (eg, daily or weekly), supporting repeatable, automated data extraction without manual handling. In many cases, the extracted datasets are further processed through ETL pipelines to harmonize field names, apply validation rules, and align data structures with the registry schema, ensuring interoperability. ETL pipelines automate the process of moving data from source systems like EMRs into target systems such as registries by first extracting relevant data, then transforming it to meet predefined standards (eg, renaming fields, standardizing units, and applying validation rules), and finally loading it into the registry database in the correct format.

Built-in EMR functionalities, such as Epic Clarity and embedded data mart solutions, were used for automated routine extraction and transfer by Milinovich and Kattan [41] (Epic Clarity, weekly extraction), Nathan et al [45] (Epic Clarity, daily extraction), Mou et al [43] (Epic Clarity, daily extraction), and Salati et al [49] (built-in EMR functionality, near real-time updates).

For unstructured or mixed-format data sources, natural language processing (NLP) methodologies were applied to automate the extraction of clinical information from free-text documentation. NLP automates the extraction of information from unstructured free-text fields in EMRs by converting narrative data into structured formats suitable for registry ingestion.

Two common NLP techniques are named entity recognition (NER) and pattern matching. NER algorithms identify and classify clinical concepts, such as diagnoses, medications, or procedures, often mapping them to standardized terminologies such as *ICD-10* (*International Statistical Classification of Diseases and Related Health Problems, Tenth Revision*) or SNOMED-CT (Systematized Nomenclature of Medicine - Clinical Terms), enabling consistent structuring of extracted data. Pattern matching, by contrast, uses predefined rules or regular expressions to detect specific text patterns (eg, medication names, dosages, and dates) without requiring machine learning models.

In EMR-to-registry integration, these methods allow clinically relevant information to be automatically detected, extracted, and formatted into predefined registry fields, reducing the need for manual chart review and enabling large-scale, systematic use of free-text clinical documentation. Bacchi et al [38] explicitly described using Python-based NLP libraries to extract stroke metrics, while Heider et al [39] reported use of Apache Unstructured Information Management Architecture for COVID-19 data extraction, with daily updates. Munzone et al [44] used a rule-based NLP algorithm, and Mou et al [42] used open- and closed-source large language models (LLMs) for oncology data extraction. Tavabi et al [59] developed a lightweight NLP pipeline to build orthopedic registries from free-text clinical notes. Wang et al [50] deployed a LLM (ChatGLM) to automate real-world data extraction in Chinese hospital settings.

Several studies extracted both structured and unstructured data, using a combination of technologies [18,22,37,40-42,50,52,62,64]. Two studies [49,54] did not clearly specify the technologies used for data extraction. One study [62] described the use of a centralized repository for aggregation of primary care data, but did not detail the specific technological mechanisms for extraction and transfer.

Middleware and standardized interoperability frameworks were also used in many studies. Nakagawa et al [61] and Sugiyama et al [60] used the Standardized Structured Medical Information eXchange version 2 (SS-MIX2) middleware framework to enable standardized extraction across hospital systems. SS-MIX2 is a standardized middleware framework developed in Japan that enables the extraction, storage, and sharing of structured clinical data from EMRs by converting it into a unified format, supporting interoperability across different health care systems. In terms of interoperability frameworks, Cheng et al [53] used REDCap Clinical Data Interoperability Services (CDIS) with daily data transfer via a FHIR (Fast Healthcare Interoperability Resources)-based application programming interface (API). CDIS enables automated extraction of structured clinical data from EMRs by packaging information into standardized FHIR bundles. FHIR organizes health care data into modular, machine-readable resources, while APIs provide the secure connection that transmits these resources to the target registry.

Technologies used to facilitate the transfer of data onto clinical registries varied across studies, with APIs and built-in EMR functionalities being the most commonly reported. Direct API integrations (eg, FHIR APIs) were reported in Stevens et al [52], Cheng et al [53], and Goel et al [58] to support structured and secure registry population, while Pittman et al [20] leveraged the Informatics for Integrating Biology & the Bedside (i2b2) middleware platform for scheduled weekly updates via an API. In other studies, such as Pan et al [46] and Salati et al [49], the specific transfer protocol was not explicitly stated; however, the use of embedded EMR functions suggests that internal secure batch transfers were used.

The frequency of data transfer varied depending on the system architecture, institutional requirements, and operational considerations, ranging from near real-time updates to intervals as long as 6 months. Near real-time updates were achieved in studies such as Abu-Rish Blakeney et al [37], Bodagh et al [14], and Salati et al [49], particularly where SQL or built-in EMR pipelines were leveraged. Daily transfers were reported by Nathan et al [45], Mou et al [43], and Heider et al [39]. Weekly or monthly batch processes were described in Kannan et al [21] and Nasir et al [55], while longer biannual update cycles were used in Garies et al [62], reflecting resource constraints. One study customized the frequency of data transfer based on an assigned schedule (eg, daily, weekly, or monthly) for each patient [52]. Twelve studies [24,38,43,44,48,50,51,56-60] did not specify the frequency of transfers used, and 17 studies [18,38,39,41,43,44,46-48,50,54,56,57,59,62-64] did not explicitly detail the technology used to transfer data onto the clinical registry.

### Evaluating Data Quality in Automated EMR-to-Registry Technologies

Data quality was assessed over three key domains: the completeness of data input, the accuracy of data extracted, and the semantic consistency of data transferred (Table S1 in Multimedia Appendix 5) [65]. Twenty-one studies [19-22,24,37,38,40,43,45-50,55,57,58,60,62,63] evaluated the completeness of data inputted into EMR systems. Among these, 13 studies [19-21,24,45-48,55,57,58,60,63] extracted exclusively from structured formats, 5 studies [22,37,40,50,62] derived data from mixed formats, 2 studies [38,43] derived data from unstructured formats, and 1 study [49] did not specify the data format. The most common methods of assessing data completeness post input were manual validation, completion rate assessments, and use of mandatory fields within structured data capture tools. Of these studies, only 8 quantified completeness of data input as a percentage, with completeness ranging from 50% to 100% [19,20,24,40,43,48-50]. One study [50] presented completeness between structured and unstructured formats, finding 100% completeness for structured data versus less than 20% for unstructured data. The remaining studies did not report completeness of data input.

Thirty studies [18-21,23,24,37-50,52,54-57,59-63] evaluated the accuracy of technology-driven data extraction. Of these, only 10 studies [19,20,23,40,43,44,49,50,52,56] expressed accuracy as a percentage, all demonstrating performance exceeding 90%, irrespective of data format. One study [43] further assessed accuracy using sensitivity and specificity metrics, while another compared accuracy of data extraction between open- and closed-source large language models [56]. Manual validation emerged as the predominant method for assessing extraction accuracy.

Several studies described alternative methods of assessing data quality that did not align with the predefined metrics summarized in Table S1 in Multimedia Appendix 5. One study applied a qualitative research-grade rating, incorporating both accuracy and completeness, without explicitly reporting percentages [37]. One study used rejection rates of XML files due to validation errors (eg, missing or inaccurate data) as an indicator of data quality [63]. Several studies used mandatory fields within structured data capture forms, implying full completeness of data input [21,22,45,58]. Three studies [20,46,48] reported data quality at the variable or domain level, such as the availability of key laboratory values or the presence of diagnosis codes, rather than providing comprehensive dataset-level metrics. In one study, data quality was assessed through subjective user perception rather than an objective measurement [55].

In addition, a variety of methods were used to maintain semantic consistency post transfer into the clinical registry. Of the 21 studies [21-23,37,38,40-43,45-50,52-58,60-64] that reported a method, the most commonly used approach was the application of standardized terminologies and coding systems. This method was reported in 11 studies [23,41,43,48,52,54,55,58,60-62], and most frequently involved *ICD-10*, Logic Observation Identifiers Names and Codes (LOINC), and SNOMED CT to ensure the consistent interpretation of clinical terms and concepts across systems. Six studies [23,37,40,55,56,63] implemented a shared data dictionary to define data elements and align their definitions across systems. Five studies [50,53,60,61,64] adhered to data exchange standards, notably Health Level 7, FHIR, CDISC, and JOIA, which define the structure and protocols for encoding and transmitting data between systems to preserve syntactic and semantic integrity. Three studies [21,42,43] applied data models to maintain structural and relational consistency across datasets. One study used standardized nomenclature [22], and 5 studies [23,43,55,60,61] used multiple approaches. The remaining 15 studies [14,18-20,24,38,39,44-47,49,51,57,59] did not specify a method for maintaining semantic consistency post transfer.

### Approaches to Data Privacy, Security, and Regulatory Compliance

To uphold privacy standards, patient de-identification prior to registry transfer was conducted in 19 studies [18,20,22,23,37,40,43,44,50,54-58,60-64] (Table S1 in Multimedia Appendix 6). Among these, 15 studies [18,20,37,40,43,44,50,54-58,61,63,64] did not report the method used. The remaining studies used various approaches, including pattern-matching algorithms [62], automated server-based anonymization [60], use of an EHR-R-REDCap pipeline [23], and manual redaction [22].

Reported security measures included the use of secure transmission protocols between EMRs and registries, infrastructure and storage protections, and access controls to safeguard registry data. The majority of studies (n=27) [14,18-24,37,38,40-45,47-55,57,59] did not report a secure transfer method. Seven studies [56,58,60-64] reported transferring the data over HTTPS, secure file transfer protocol, or virtual private network, and 2 studies [39,46] reported applying security protocols such as secure sockets layer to encrypt and protect data during transfer. Two further studies [37,53] reported using a secure encrypted channel without specifying the method.

Nine studies [22,37,44,53,57,60,62-64] adopted secure infrastructure and storage measures to protect registry data. These included Health Insurance Portability Accountability Act (HIPAA)–compliant storage (n=3) [22,37,53], centralized secure housing (n=2) [60,62], secure cloud infrastructure (n=2) [44,64], an intranet-based system (n=1) [57], and a secure web-based interface (n=1) [63]. One study stated registry storage was secure, but did not specify the measures taken [61].

Methods used to control and authenticate access to registry data included audit logs (n=4) [46,53,62,63], role-based access control (n=3) [20,53,63], multifactor authentication (MFA) (n=2) [22,44], password and USB key security (n=2) [57,62], token-based access control (n=1) [53], single sign-on (n=1) [22], and permission set access control (n=1) [40]. While some studies implemented multiple measures (n=6) [22,39,53,57,62,63], others restricted access without specifying the methods used (n=8) [37,42,47,52,55,60,61,64].

Various measures were implemented to ensure registry regulatory compliance, including participant consent procedures, ethical oversight, restrictions on data use, and adherence to regulatory standards (Table S1 in Multimedia Appendix 7). Consent procedures were reported in 9 studies [40,42,44,55-57,60-62], including a waiver of consent (n=5) [40,42,55-57], opt-out procedures (n=3) [60-62], and explicit opt-in consent (n=1) [44]. Ethics approval for the registry was obtained in 23 studies [14,19,20,23,24,37,38,40,44,46,47,50,52,53,55-57,59-64], with one study receiving an exception [42]. To restrict registry data use, institutional review board (IRB) oversight (n=19) [20,21,23,24,37,40,42,46,47,50,53,55-57,59-62,64], research approval processes (n=8) [40,44,53,60-64], and data sharing agreements (n=5) [21,37,44,62,63] were used.

Only a few studies referenced compliance with jurisdiction-specific regulatory standards. Of the studies conducted in the United States, only 4 explicitly reported adherence to the HIPAA [22,37,53,55]. One study based in Japan cited compliance with the Act on the Protection of Personal Information (APPI) [64], while one European study referenced alignment with the General Data Protection Regulation [44]. The remaining 30 studies [14,18-21,23,24,38-43,45-52,54,56-63] did not reference adherence to a jurisdiction-specific regulatory standard, though some demonstrated efforts to ensure data privacy and security, suggesting alignment with regulatory principles.

### Implementation Challenges and Frameworks for Clinical Data Technologies

Thirty-four studies identified a range of challenges associated with implementation (Table S1 in Multimedia Appendix 8) [14,18-24,37-40,42-56,58-64]. Each challenge aligned with at least one of the identified thematic categories: (1) data quality, (2) data mapping, standardization and semantic harmonization, (3) technical, infrastructure and resource availability, (4) workflow integration and adoption, (5) interoperability and data integration across systems, (6) privacy, security and governance, (7) registry scope, generalizability and maintenance, and (8) semantic and temporal issues. The most commonly reported challenges included missing or incomplete data [18,19,38,40,47,55], inaccurate data input or extraction [42-44,49-51], significant time and resource demands associated with system development and implementation [23,24,50,54,56,59], poor staff buy-in, uptake, or adoption [14,21,45,47,53], and the inability to capture events occurring outside the institution [18,37,40,44,55]. One study broadly highlighted technical, policy, and governance challenges without providing specific details [53].

Despite the breadth and volume of challenges identified, no studies explicitly reported the use of an implementation science framework to guide technological development and implementation. Instead, 3 studies referenced the use of formal software development methodologies, specifically the Agile Methodology [14,21] and a modified Software Development Life Cycle [37]. An additional 6 studies incorporated elements commonly aligned with implementation science frameworks, including iterative development and co-design, stakeholder involvement and collaborative problem-solving, embedded feedback loops, and rapid-cycle improvement processes [45,49,53,56,63,64].

### Mapping Evidence and Contextual Factors for EMR-to-Registry Interoperability

**Figure 3.**
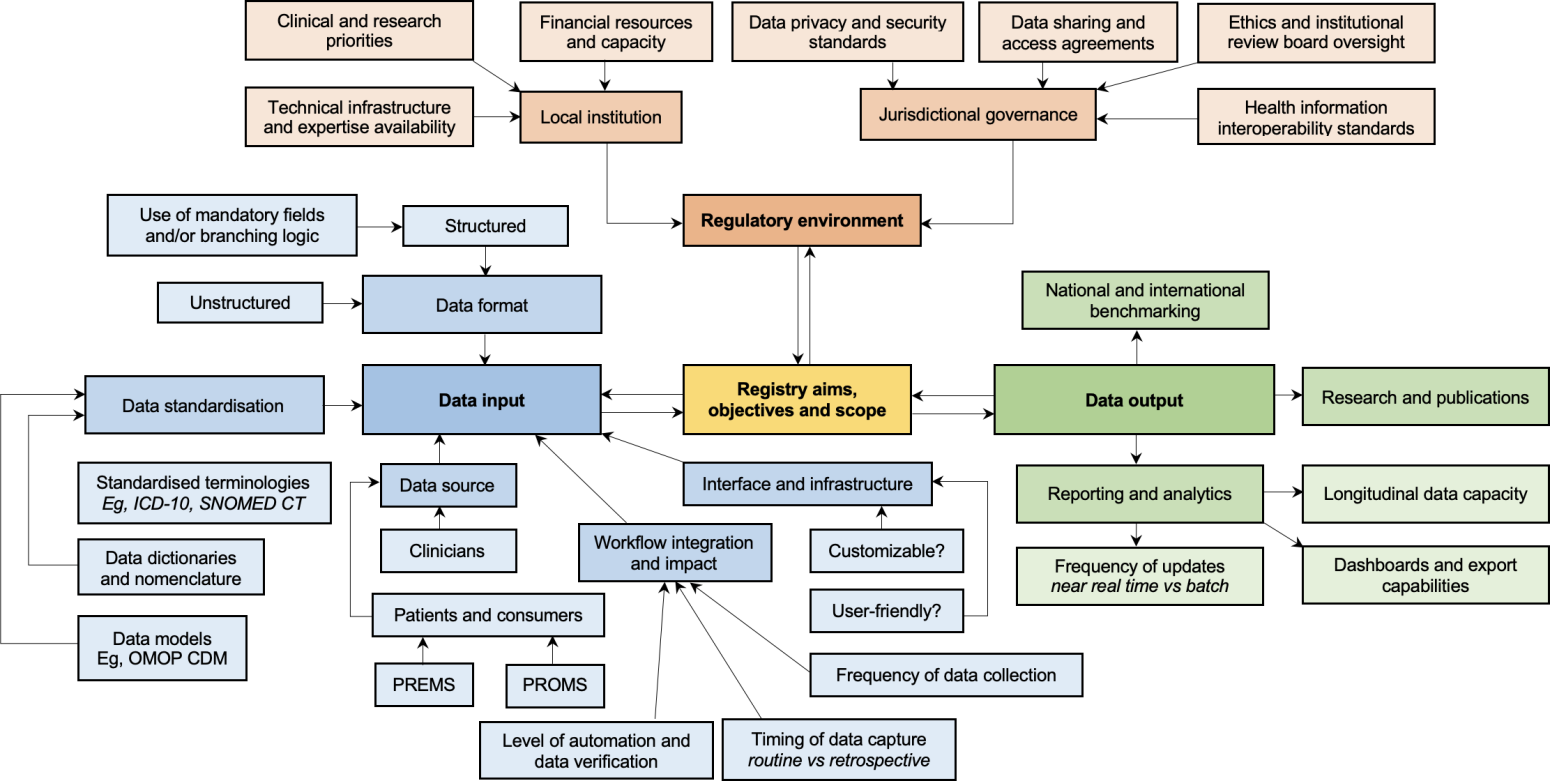
Key contextual considerations influencing technology selection for electronic medical record-to-registry interoperability. *ICD-10*: *International Statistical Classification of Diseases and Related Health Problems, Tenth Revision*; OMOP CDM: observational medical outcomes partnership common data model; PREM: patient reported experience measures; PROM: patient reported outcome measures; SNOMED CT: Systematized Nomenclature of Medicine - Clinical Terms.

To understand the current landscape of EMR-to-registry integration, evidence on reported technologies, data formats, and implementation challenges was synthesized. Table S1 in Multimedia Appendix 9 presents an evidence gap map summarizing these practices, highlighting the heterogeneity of solutions implemented across studies and the prevalence of implementation challenges, revealing areas that require targeted improvement to support successful integration. Importantly, the map also underscores gaps in reporting technologies used to transfer extracted data from EMRs to clinical registries, a critical component for achieving interoperability. These findings are contextualized in Figure 3, which provides a consolidated overview of factors influencing technological selection, development, and implementation.

## Discussion

### Principal Findings

This review advances understanding of interoperability between EMRs and clinical registries by uniquely examining automated and sustainable solutions for data exchange, extending beyond prior work that has largely focused on technologies designed for isolated or study-specific data extraction. Through a synthesis of studies examining existing technological approaches, this review reveals that current approaches are highly variable, context-dependent, and inconsistently evaluated across studies. While existing studies provide important insight into how these processes can be tailored to meet the unique needs and constraints of each health care context, the absence of consistent quality reporting, robust evaluation, and methodological rigor meant no single approach could be recommended. Notably, none of the included studies used a rigorous or comparative study design to evaluate system effectiveness or impact, with most relying on descriptive approaches. This is further compounded by inconsistent and non-standardized reporting on data quality metrics, privacy, security, and regulatory compliance processes, as well as limited application of implementation science frameworks despite reported challenges. Collectively, these limitations restrict meaningful comparisons across studies, and by extension, the technological insights that could inform future implementation.

A novel contribution of this review was the synthesis of the context-specific considerations that influenced technology selection, as informed by reported implementations in the literature and summarized in Figure 3. This synthesis revealed that a registry’s purpose and scope not only shaped decisions regarding what data was collected (input) and how it was structured, managed, and used (output), but was itself reciprocally influenced by the broader institutional and jurisdictional regulatory environment. Collectively, these interrelated factors not only influenced the feasibility of implementation, but also had profound implications for data quality, degree of automation and interoperability, scalability, and long-term sustainability. These insights have direct implications for future implementations of interoperable technologies, guiding strategic design and governance decisions that promote sustainability, security, and regulatory compliance, while enabling the ethical and efficient reuse of health data for clinical care, quality improvement, and research.

One key factor influencing both the selection and performance of technologies was the data format, ranging from highly structured fields to unstructured free text. Most studies extracted data from structured formats, benefiting from their standardization, compatibility with coding systems, and designated entry fields that enhance data quality and completeness, especially when supported by mandatory fields and branching logic. However, more than 80% of clinical data exists in unstructured text [12], which remains underused, highlighting the missed potential for richer data capture and integration. Unlike structured fields, free text enables nuanced narrative expressivity in clinical documentation, making their effective use crucial for comprehensive patient records and research insights [66]. While some studies used NLP for unstructured data extraction, wider adoption and continued advancement of artificial intelligence (AI)–driven techniques could further enhance the ability to extract meaningful insights from such data, ultimately improving both clinical decision-making and research potential [67].

A key consideration in EMR research is the widespread concern regarding the reliability and usability of EMR-derived data for clinical research, as noted in previous literature [68-70]. The challenge remains in using an imperfect system, originally designed to support clinical care, financial billing, and insurance claims, for clinical research [8]. Missing data, varying levels of standardization, and a lack of harmonized terminologies can introduce the risk of biases, limit reproducibility, and hinder the integration of EMR-derived data into high-quality research and evidence-based practice. A balancing axiom suggests that data fields actively used, frequently accessed, and regularly reviewed in clinical workflows are more likely to demonstrate improved quality and reliability over time [21]. However, routine clinical use alone does not guarantee data quality and therefore does not negate the need for standardized and transparent evaluation practices. Addressing these foundational issues is essential to improving the credibility, use, and long-term value of EMR-linked registry systems in high-quality clinical research.

At a minimum, studies should report key data quality metrics such as completeness of data input into EMR systems, accuracy of data extracted using technological methods, and semantic consistency of data transferred onto registry systems. The use of existing frameworks which aim to assess data quality [71-73] and semantic consistency [6,74] can help standardize these processes and enhance methodological rigor. Where appropriate, these metrics should also be reported using quantifiable measures (eg, the percentage of missing data) and be accompanied by a clear description of the methods used to calculate them. This ensures that technologies in future research can be evaluated consistently, enabling reliable cross-study comparisons and supporting the development of safe, transparent, and evidence-based recommendations for improving EMR-to-registry interoperability.

Alongside data quality concerns, inconsistent reporting on measures ensuring data privacy, security, and regulatory compliance highlight additional issues surrounding patient confidentiality and the protection of their sensitive information. While these registries enable collaborative data use that enhances both research and clinical outcomes which reciprocally benefit patients, they introduce legal and ethical considerations regarding patient rights and consent, particularly when data is repurposed for research without explicit authorization [75-77]. Common use of opt-out procedures and ethical waivers of consents, particularly when de-identified data is used, has facilitated large-scale data access for research while minimizing direct patient burden [78,79]. However, these approaches raise ethical concerns pertaining to transparency, patient autonomy, and privacy risks, especially when the distinction between de-identified and re-identifiable data has not been clearly articulated [76]. True de-identification is not possible in registries where data pertaining to the same patient is entered at multiple timepoints. In such cases, pseudonymization is commonly used, replacing patient identifiers with coded keys that enable longitudinal linkage of records while preserving patient confidentiality. The secure management of these re-identification keys is therefore a crucial governance consideration, balancing the need for data use in longitudinal research with the ethical imperative to protect patient privacy and autonomy. Notably, no patient perspectives were captured in any of the studies reviewed, revealing a significant gap regarding patient experiences and their views on the reuse of their data in clinical registries [80]. Similarly, inconsistencies in the use of secure transfer methodologies and poor reporting of jurisdiction-specific regulatory compliance highlight potential risks in integrating and harmonizing data from EMRs to clinical registries. These issues underscore the critical necessity for improved transparency and rigorous adherence to governance frameworks to ensure that data exchange between EMRs and clinical registries remains secure, ethical, and aligned with regulatory standards across diverse health data ecosystems.

Given the sensitivity of health information, technical safeguards must be in place to maintain patient privacy, comply with regulatory requirements, and build trust among stakeholders. Several established and emerging technical solutions are being used to address these concerns. Encryption, both at rest and in transit, is a foundational measure that ensures data cannot be accessed or intercepted without authorization [81]. Role-based access controls and audit logging further enhance security by restricting access to authorized personnel and maintaining traceability of data interactions [82]. In some cases, federated data models and privacy-preserving record linkage allow for data analysis across institutions without centralizing identifiable information, thereby minimizing exposure [83]. The use of secure cloud infrastructure, compliant with standards such as ISO 27001, HIPAA, and General Data Protection Regulation, has also become more prevalent, offering scalable and resilient environments for data storage and processing [81]. Additionally, data governance frameworks play a critical role in defining policies for data access, sharing, and reuse, ensuring that ethical and legal considerations are upheld [84]. Emerging technologies such as homomorphic encryption and differential privacy offer promising avenues for enabling secure computation on sensitive data without compromising individual privacy [85]. While these approaches are still maturing, they represent important innovations in the field of health data protection.

Efforts to improve data quality and regulatory compliance must also minimize clinical burden [86,87]. The variety of implementation challenges reported across studies highlights the complexity of integrating these tools into clinical and registry workflows, with common barriers including data quality concerns, limited staff engagement, high resource demands for system development, and poor alignment with existing institutional infrastructure and workflows. Despite these challenges, there was a paucity of studies explicitly applying an implementation science framework. This theoretical gap likely contributes to the fragmented nature of reporting and hinders the successful adoption, integration, and long-term sustainability of EMR to registry integration. The use of process-driven models and determinant frameworks is a well-established antecedent to successful technology adoption [88-91]. A multidisciplinary, co-design, implementation-informed approach is needed to support alignment with clinical workflows and maximize research use while minimizing disruptions to patient care [92,93].

While automated technologies offer significant advantages over manual data entry, their feasibility is heavily dependent on local financial and technical capacity. Most studies originated from high-income countries, with mature EMR systems and infrastructure. In contrast, low-middle income countries reportedly faced persistent barriers stemming from limited funding and infrastructure capacity [19,63]. The implementation of EMRs and the subsequent development and maintenance of clinical registries is well recognized as a costly and complex process, requiring substantial investment in system design, infrastructure setup, maintenance, staff training, data security, and interoperability [94-96]. Beyond the initial setup, registries also incur ongoing costs that are not typically funded by traditional research project grants, adding an additional element of complexity to their implementation and long-term sustainability across organizations. This presents a major barrier for low-middle income countries, where differences in health system models, funding structures, and technological infrastructure further limit the feasibility of widespread EMR integration. Even in high-income countries such as Australia, the roll-out of interoperable registries is further complicated by the fact that most states have adopted different EMR solutions with varying levels of implementation across hospitals, despite the availability of funding. These disparities threaten to widen global gaps in clinical research capacity, data-driven decision-making, and ultimately patient outcomes.

### Limitations of This Study

This review has several limitations. Heterogeneity in study designs and inconsistent reporting of technology characteristics constrained the potential for direct comparison and meta-analysis. Only English studies published between January 2013 and April 2025 were included. Excluding gray literature may result in the underrepresentation of relevant real-world technological implementations, particularly outside high-income countries, and could potentially bias findings toward well-resourced settings. Nonindexed sources may also have been missed as they were not systematically searched outside the selected databases. Technologies designed for single study use were also excluded, which may have provided additional insights. Study authors were not contacted for clarification, and all interpretations relied solely on the information presented in the published manuscripts. Furthermore, many included studies are situated within specific national contexts (eg, SS-MIX2 in Japan), which may limit the broader international generalizability of the findings.

### Conclusions

This review provides insight into the diverse strategies that facilitate interoperability between EMRs and clinical registries. While technological advancements have negated the need for manual data extraction and transfer onto clinical registries, challenges remain in ensuring access, consistency, security, and sustainability across diverse health care settings. Given the ethical, legal, and regulatory complexities of reusing clinical data for research, future initiatives must operate within robust governance frameworks to safeguard data security, protect patient privacy, and ensure compliance with both institutional and jurisdictional policies while promoting transparency, accountability, and equitable access to research opportunities. Implementation efforts should be tailored to local contexts, guided by implementation science frameworks and informed by meaningful engagement with end users to support adoption. Overcoming these barriers, alongside continued innovation in interoperability solutions, will be essential to maximizing the potential and impact of EMR-linked registries to drive high-quality research, strengthen clinical decision-making, and ultimately improve patient outcomes.

## Supplementary material

10.2196/82380 Multimedia Appendix 1 Search terms and strategy.

10.2196/82380 Multimedia Appendix 2 Data extraction categories and description.

10.2196/82380 Multimedia Appendix 3 Participant and study characteristics of included studies.

10.2196/82380 Multimedia Appendix 4 Summary of electronic medical record and registry infrastructure, technologies, and interoperability approaches.

10.2196/82380 Multimedia Appendix 5 Summary of data quality measures following data input, extraction, and transfer.

10.2196/82380 Multimedia Appendix 6 Summary of data privacy and security measures.

10.2196/82380 Multimedia Appendix 7 Summary of measures for registry regulatory compliance.

10.2196/82380 Multimedia Appendix 8 Summary of implementation challenges identified in establishing electronic medical record-to-registry interoperability.

10.2196/82380 Multimedia Appendix 9 Evidence gap map of technologies, data formats, and implementation challenges in electronic medical record-to-registry integration.

10.2196/82380 Checklist 1 PRISMA-ScR checklist.

## Abbreviations

AI: artificial intelligence
API: application programming interface
APPI: Act on the Protection of Personal Information
CDIS: Clinical Data Interoperability Services
CDISC: Clinical Data Interchange Standards Consortium
CDISC: Clinical Data Interchange Standards Consortium
EHR: electronic health record
EMR: electronic medical record
ETL: extract, transform, load
FHIR: Fast Healthcare Interoperability Resources
HIPAA: Health Insurance Portability and Accountability Act
i2b2: Informatics for Integrating Biology & the Bedside
*ICD-10*: *International Statistical Classification of Diseases and Related Health Problems, Tenth Revision*
IRB: institutional review board
LLM: large language model
LOINC: Logic Observation Identifiers Names and Codes
MFA: multifactor authentication
NER: named entity recognition
NLP: natural language processing
PRISMA: Preferred Reporting Items for Systematic Reviews and Meta-Analyses
PRISMA-ScR: Preferred Reporting Items of Systematic Reviews and Meta-Analyses extension for Scoping Reviews
REDCap: Research Electronic Data Capture
SNOMED CT: Systematized Nomenclature of Medicine – Clinical Terms
SQL: structured query language
SS-MIX2: Standardized Structured Medical Information exchange version 2
