# Supplementary material for: Exploring Technological Solutions for Interoperability Between Patient Electronic Medical Records and Clinical Registries: Scoping Review
Source: J Med Internet Res. 2026 May 25;28:e82380. doi: 10.2196/82380 (PMC13200772; doi:10.2196/82380)
Supplement: Multimedia Appendix 1 [file jmir-v28-e82380-s001.docx]

**Table S1.** Search terms and strategy.

| ***Database*** | ***Search String*** |
| --- | --- |
| PubMed | ("Registries"[Mesh] OR “Translational Research, Biomedical"[Mesh] OR “Health Services Research"[Mesh] OR "registries"[tiab] OR "registry"[tiab] OR “repository”[tiab] OR “health information”[tiab] OR “health data”[tiab] OR “health management”[tiab] OR "Clinical database*"[tiab] OR "Clinical database*"[tiab] OR "Patient database*"[tiab] OR "Patient database*"[tiab])  AND  ("Electronic Health Records"[Mesh] OR "electronic health record*"[tiab] OR "electronic medical record*"[tiab] OR "EHR"[tiab] OR "EMR"[tiab] OR "IEMR"[tiab] OR "Computerized Medical Record*"[tiab] OR "Computerised Medical Record*"[tiab] OR "cerner"[tiab] OR "epic"[tiab])  AND  ("Information Storage and Retrieval"[Mesh] OR “Artificial intelligence” [Mesh] OR “Health information interoperability” [Mesh] OR retrieval[tiab] OR "record link*"[tiab] OR abstraction[tiab] OR interoperability [tiab] OR “inter-operability”[tiab] OR linkage*[tiab] OR extract[tiab] OR extracting[tiab] OR extracted[tiab] OR linked[tiab] OR capture[tiab] OR automate[tiab] OR automated[tiab] OR “natural language processing”[tiab] OR “health information exchange”[tiab])  AND  (“randomized controlled trial”[pt] OR “controlled clinical trial”[pt] OR randomized[tiab] OR randomised[tiab] OR placebo[tiab] OR randomly[tiab] OR trial[tiab] OR groups[tiab] OR Crossover[tiab] OR "Comparative Study"[pt] OR "Evaluation Study"[pt] OR "Epidemiologic Studies"[Mesh] OR “case-control studies”[Mesh] OR “Cohort Studies”[Mesh] OR “case control”[tiab] OR Cohort[tiab] OR “Follow up”[tiab] OR Observational[tiab] OR Longitudinal[tiab] OR Prospective[tiab] OR retrospective[tiab] OR “cross sectional”[tiab] OR “Cross-Sectional Studies”[Mesh] OR Investigated[tiab] OR Evaluated[tiab] OR Impact[tiab] OR Analysis[tiab] OR Statistics[tiab] OR Data[tiab] OR "statistics and numerical data"[sh] OR "epidemiology"[sh]) |
| CINAHL | ((MH "Information Systems+”) OR (MH "Translational Research") OR (MH "Health Services Research+") OR (TI registries OR AB registries) OR (TI registry OR AB registry) OR (TI repository OR AB repository) OR (TI "health information" OR AB "health information") OR (TI "health data" OR AB "health data") OR (TI "health management" OR AB "health management") OR (TI "Clinical database*" OR AB "Clinical database*") OR (TI "Clinical database*" OR AB "Clinical database*") OR (TI "Patient database*" OR AB "Patient database*") OR (TI "Patient database*" OR AB "Patient database*"))  AND  ((MH "Electronic Health Records+") OR (TI "electronic health record*" OR AB "electronic health record*") OR (TI "electronic medical record*" OR AB "electronic medical record*") OR (TI EHR OR AB EHR) OR (TI EMR OR AB EMR) OR (TI IEMR OR AB IEMR) OR (TI "Computerized Medical Record*" OR AB "Computerized Medical Record*") OR (TI "Computerised Medical Record*" OR AB "Computerised Medical Record*") OR (TI cerner OR AB cerner) OR (TI epic OR AB epic))  AND  ((MH "Electronic Data Interchange+") OR (MH "Information Retrieval+") OR OR (MH "Artificial intelligence+") OR (MH "Health information interoperability+") OR (TI retrieval OR AB retrieval) OR (TI "record link*" OR AB "record link*") OR (TI abstraction OR AB abstraction) OR (TI interoperability OR AB interoperability) OR (TI inter-operability OR AB inter-operability) OR (TI linkage* OR AB linkage*) OR (TI extract OR AB extract) OR (TI extracting OR AB extracting) OR (TI extracted OR AB extracted) OR (TI linkedOR AB linked) OR (TI capture OR AB capture) OR (TI automate OR AB automate) OR (TI automated OR AB automated) OR (TI "natural language processing" OR AB "natural language processing") OR (TI "health information exchange" OR AB "health information exchange"))  AND  ((MH "Clinical Trials+") OR (MH "Quantitative Studies") OR TI placebo* OR AB placebo* OR (MH "Placebos") OR (MH "Random Assignment") OR TI random* OR AB random* OR TI ((singl* or doubl* or tripl* or trebl*) W1 (blind* or mask*)) OR AB ((singl* or doubl* or tripl* or trebl*) W1 (blind* or mask*)) OR TI clinic* trial* OR AB clinic* trial* OR PT clinical trial) OR ((MH "Epidemiological Research+") OR (MH "Case Control Studies+") OR (MH "Prospective Studies+") OR (TI "case control" OR AB "case control") OR (TI Cohort OR AB Cohort) OR (TI "Follow up" OR AB "Follow up") OR (TI Observational OR AB Observational) OR (TI Longitudinal OR AB Longitudinal) OR (TI Prospective OR AB Prospective) OR (TI retrospective OR AB retrospective) OR (TI "cross sectional" OR AB "cross sectional") OR (MH "Cross Sectional Studies+") OR (TI Investigated OR AB Investigated) OR (TI Evaluated OR AB Evaluated) OR (TI Impact OR AB Impact) OR (TI Analysis OR AB Analysis) OR (TI Statistics OR AB Statistics) OR (TI Data OR AB Data)) |
| Embase | (‘Patient Registry’/exp/mj OR 'Translational Research'/exp/mj OR 'Health Services Research'/exp/mj OR ‘Medical Research’/exp/mj OR registries:ti,ab OR registry:ti,ab OR repository:ti,ab OR 'health information':ti,ab OR 'health data':ti,ab OR 'health management':ti,ab OR 'Clinical database*':ti,ab OR 'Clinical database*':ti,ab OR 'Patient database*':ti,ab OR 'Patient database*':ti,ab)  AND  ('Electronic Health Records'/exp/mj OR 'electronic health record*':ti,ab OR 'electronic medical record*':ti,ab OR EHR:ti,ab OR EMR:ti,ab OR IEMR:ti,ab OR 'Computerized Medical Record*':ti,ab OR 'Computerised Medical Record*':ti,ab OR cerner:ti,ab OR epic:ti,ab)  AND  ('Information Storage and Retrieval'/exp/mj OR 'Information Processing'/exp/mj OR 'data processing'/exp/mj OR retrieval:ti,ab OR 'record link*':ti,ab OR abstraction:ti,ab OR interoperability:ti,ab OR inter-operability:ti,ab OR linkage*:ti,ab OR extract:ti,ab OR extracting:ti,ab OR extracted:ti,ab OR linked:ti,ab OR capture:ti,ab OR automate:ti,ab OR automated:ti,ab OR 'natural language processing':ti,ab OR 'health information exchange':ti,ab)  AND  (random* OR factorial OR crossover OR placebo OR blind OR blinded OR assign OR assigned OR allocate OR allocated OR 'crossover procedure'/exp OR 'double-blind procedure'/exp OR 'randomized controlled trial'/exp OR 'single-blind procedure'/exp OR 'epidemiology'/exp OR 'controlled study'/exp OR 'cohort analysis'/exp OR "case control":ti,ab OR Cohort:ti,ab OR "Follow up":ti,ab OR Observational:ti,ab OR longitudinal:ti,ab OR Prospective:ti,ab OR retrospective:ti,ab OR "cross sectional":ti,ab OR 'Cross-Sectional Studies'/exp OR Investigated:ti,ab OR Analysis:ti,ab OR Statistics:ti,ab OR Data:ti,ab) |
| Web of Science | (TI=("registries" OR "registry" OR “repository” OR “health information” OR “health data” OR “health management” OR "Clinical database*" OR "Clinical database*" OR "Patient database*" OR "Patient database*")) OR AB=("registries" OR "registry" OR “repository” OR “health information” OR “health data” OR “health management” OR "Clinical database*" OR "Clinical database*" OR "Patient database*" OR "Patient database*")  AND  (TI=("electronic health record*" OR "electronic medical record*" OR "EHR" OR "EMR" OR "IEMR" OR "Computerized Medical Record*" OR "Computerised Medical Record*" OR "cerner" OR "epic")) OR AB=("electronic health record*" OR "electronic medical record*" OR "EHR" OR "EMR" OR "IEMR" OR "Computerized Medical Record*" OR "Computerised Medical Record*" OR "cerner" OR "epic")  AND  (TI=(retrieval OR "record link*" OR abstraction OR interoperability OR “inter-operability” OR linkage* OR extract OR extracting OR extracted OR linked OR capture OR automate OR automated OR “natural language processing” OR “health information exchange”)) OR AB=(retrieval OR "record link*" OR abstraction OR interoperability OR “inter-operability” OR linkage* OR extract OR extracting OR extracted OR linked OR capture OR automate OR automated OR “natural language processing” OR “health information exchange”)  AND  (Trial OR randomized OR randomised OR placebo OR randomly OR groups OR "Epidemiologic Studies" OR Epidemiological OR "case-control studies" OR "Cohort Studies" OR "case control" OR Cohort OR "Follow up" OR Observational OR longitudinal OR Prospective OR retrospective OR "cross sectional" OR "Cross-Sectional Studies" OR Investigated OR Analysis OR Statistics OR Data) |
| Year Limit | January 2013 - April 2025 |
