# Supplementary material for: Exploring Technological Solutions for Interoperability Between Patient Electronic Medical Records and Clinical Registries: Scoping Review
Source: J Med Internet Res. 2026 May 25;28:e82380. doi: 10.2196/82380 (PMC13200772; doi:10.2196/82380)
Supplement: Multimedia Appendix 2 [file jmir-v28-e82380-s002.docx]

**Table S1.** Data extraction categories and description.

| **Category** | **Description** |
| --- | --- |
| Study Identifiers | Author, year, title |
| Study Characteristics | Single/multi-center, country, disease/specialty, registry/database name |
| System Used | Electronic medical record (EMR), clinical registry |
| Technology Details | Source format, name of technology used for extraction, name of technology used for data transfer, transfer frequency |
| Data Details | Data quality measures (completeness, accuracy and semantic consistency), data privacy and security measures (de-identification, transfer security, storage, access control) and regulatory compliance (consent procedures, ethical approval, reference to relevant regulatory standard, data usage control) |
| Implementation Approach | Identified barriers to implementation, implementation approach/framework used |
| Study Limitations | Other challenges or constraints noted |
