# Supplementary material for: Exploring Technological Solutions for Interoperability Between Patient Electronic Medical Records and Clinical Registries: Scoping Review
Source: J Med Internet Res. 2026 May 25;28:e82380. doi: 10.2196/82380 (PMC13200772; doi:10.2196/82380)
Supplement: Multimedia Appendix 3 [file jmir-v28-e82380-s003.docx]

**Table S1.** Participant and study characteristics of included studies.

| **First Author** | **Single/Multicenter *(Country)*** | **Study Design** | **Number of Patients*** | **Healthcare Setting** | **Clinical Focus** |
| --- | --- | --- | --- | --- | --- |
| Abu-Rish Blakeney et al.^1^ | Single *(USA)* | Descriptive Technical Report | 175,200 | Inpatient Care | Cardiac |
| Bacchi et al.^2^ | Single *(Australia)* | Descriptive Pilot Validation Study | 438 | Inpatient Care | Stroke |
| Bodagh et al.^3^ | Single *(UK)* | Observational Feasibility Study | 360 | Outpatient Care | Cardiac |
| Chen et al.^4^ | Single *(USA)* | Descriptive Technical Report | 12,341 | Outpatient Care | Oncology |
| Cheng et al.^5^ | Multicenter *(USA)* | Descriptive Technical Report | Not reported | Combination | Not Reported |
| Dalhatu et al.^6^ | Multicenter *(Nigeria)* | Descriptive Technical Report | 1,477,064 | Combination | HIV/Aids |
| Dong et al.^7^ | Multicenter *(China)* | Quality Improvement Study | Not reported | Combination | Stroke |
| Garies et al.^8^ | Multicenter *(Canada)* | Descriptive Technical Report | 410,951 | Combination | Primary Care |
| Goel et al.^9^ | Multicenter *(USA)* | Descriptive Technical Report | Not reported | Not Reported | Oncology |
| González et al.^10^ | Multicenter *(Spain)* | Descriptive Technical Report | Not reported | Combination | Not Reported |
| Heider et al.^11^ | Single *(USA)* | Descriptive Pilot Validation Study | Not reported | Telehealth | COVID-19 |
| Kannan et al.^12^ | Multicenter *(USA)* | Descriptive Technical Report | >60,000 | Outpatient Care | Chronic Diseases |
| Kapoor et al.^13^ | Multicenter *(USA)* | Descriptive Technical Report | Not reported | Outpatient Care | Oncology |
| Kariuki et al.^14^ | Single *(Kenya)* | Descriptive Technical Report | 3,901 | Outpatient Care | HIV/Aids |
| Li et al.^15^ | Single *(China)* | Descriptive Technical Report | 295,297 | Not Reported | Hypertension |
| Milinovich et al.^16^ | Single *(USA)* | Descriptive Technical Report | Not Reported | Not Reported | Not Reported |
| Miyake et al.^17^ | Multicenter *(Japan)* | Descriptive Technical Report | Not Reported | Not Reported | Ophthalmology |
| Mou et al.^18^ | Single *(USA)* | Descriptive Pilot Validation Study | 1,717 | Not Reported | Emergency Surgery |
| Mou et al.^19^ | Single *(Germany)* | Descriptive Pilot Validation Study | 50 | Not Reported | Oncology |
| Munzone et al.^20^ | Single *(Italy)* | Descriptive Pilot Validation Study | 116 | Not Reported | Oncology |
| Nakagawa et al.^21^ | Multicenter *(Japan)* | Observational Feasibility Study | 39,121 | Outpatient Care | Chronic Renal |
| Nasir et al.^22^ | Multicenter *(USA)* | Descriptive Technical Report | 113,022 | Outpatient Care | Cardiac |
| Nathan et al.^23^ | Single *(USA)* | Descriptive Technical Report | Not Reported | Inpatient Care | Stroke |
| Pan et al.^24^ | Single *(USA)* | Descriptive Pilot Validation Study | 1,296 | Not Reported | Oncology |
| Pittman et al.^25^ | Multicenter *(USA)* | Descriptive Technical Report | 6,050 | Not Reported | Neurosurgery |
| Rayman et al.^26^ | Single *(Israel)* | Descriptive Pilot Validation Study | 104 | Surgical Care | Colorectal Surgery |
| Rubio-Mayo et al.^27^ | Single *(Spain)* | Descriptive Technical Report | 1,253 | Not Reported | Orthopedic |
| Salati et al.^28^ | Single *(Italy)* | Descriptive Pilot Validation Study | Not Reported | Surgical Care | Thoracic Surgery |
| Shalhout et al.^29^ | Multicenter *(USA)* | Descriptive Technical Report | 1,109 | Not Reported | Oncology |
| Stevens et al.^30^ | Single *(USA)* | Descriptive Pilot Validation Study | Not Reported | Combination | Syphilis Surveillance |
| Sugiyama et al.^31^ | Multicenter *(Japan)* | Descriptive Technical Report | Not Reported | Not Reported | Diabetics |
| Tavabi et al.^32^ | Multicenter *(USA)* | Descriptive Pilot Validation Study | Not Reported | Surgical Care | Orthopedic |
| Valencia Morales et al.^33^ | Multicenter *(USA)* | Descriptive Pilot Validation Study | 652 | Inpatient Care | COVID-19 |
| Wang et al.^34^ | Single *(China)* | Descriptive Pilot Validation Study | Not Reported | Not Reported | Cardiac |
| Williams et al.^35^ | Single *(USA)* | Descriptive Technical Report | Not Reported | Not Reported | Hearing Loss |
| Wulff et al.^36^ | Single *(Germany)* | Descriptive Pilot Validation Study | Not Reported | Not Reported | Pediatrics |

*(N.B. * reports the number of patients from whom data were extracted from. Studies classed as ‘Not reported” may report number of charts extracted from but did not clarify whether these represented unique patients)*

**REFERENCE LIST**

1. Abu-Rish Blakeney E, Wolpin S, Lavallee DC, Dardas T, Cheng R, Zierler B. Developing and implementing a heart failure data mart for research and quality improvement. *Inform Health Soc Care*. 2019;44(2):164-175. doi:10.1080/17538157.2018.1455202

2. Bacchi S, Gluck S, Koblar S, Jannes J, Kleinig T. Automated information extraction from free-text medical documents for stroke key performance indicators: a pilot study. *Intern Med J*. Feb 2022;52(2):315-317. doi:10.1111/imj.15678

3. Bodagh N, Archbold RA, Weerackody R, et al. Feasibility of real-time capture of routine clinical data in the electronic health record: a hospital-based, observational service-evaluation study. *BMJ Open*. 2018;8(3):e019790. doi:10.1136/bmjopen-2017-019790

4. Chen AM, Kupelian PA, Wang PC, Steinberg ML. Development of a Radiation Oncology-Specific Prospective Data Registry for Research and Quality Improvement: A Clinical Workflow-Based Solution. *JCO Clin Cancer Inform*. Dec 2018;2:1-9. doi:10.1200/cci.17.00036

5. Cheng AC, Duda SN, Taylor R, et al. REDCap on FHIR: Clinical Data Interoperability Services. *Journal of Biomedical Informatics*. 2021/09/01/ 2021;121:103871. doi:<https://doi.org/10.1016/j.jbi.2021.103871>

6. Dalhatu I, Aniekwe C, Bashorun A, et al. From Paper Files to Web-Based Application for Data-Driven Monitoring of HIV Programs: Nigeria's Journey to a National Data Repository for Decision-Making and Patient Care. *Methods Inf Med*. Sep 2023;62(3-04):130-139. doi:10.1055/s-0043-1768711

7. Dong Y, Fang K, Wang X, et al. The network of Shanghai Stroke Service System (4S): A public health-care web-based database using automatic extraction of electronic medical records. *Int J Stroke*. Jul 2018;13(5):539-544. doi:10.1177/1747493018765492

8. Garies S, Cummings M, Forst B, et al. Achieving quality primary care data: a description of the Canadian Primary Care Sentinel Surveillance Network data capture, extraction, and processing in Alberta. *Int J Popul Data Sci*. Jul 29 2019;4(2):1132. doi:10.23889/ijpds.v4i2.1132

9. Goel AK, Campbell WS, Moldwin R. Structured Data Capture for Oncology. *JCO Clin Cancer Inform*. Feb 2021;5:194-201. doi:10.1200/cci.20.00103

10. González L, Pérez-Rey D, Alonso E, et al. Building an i2b2-Based Population Repository for Clinical Research. *Stud Health Technol Inform*. Jun 16 2020;270:78-82. doi:10.3233/shti200126

11. Heider PM, Pipaliya RM, Meystre SM. A Natural Language Processing Tool Offering Data Extraction for COVID-19 Related Information (DECOVRI). *Stud Health Technol Inform*. Jun 6 2022;290:1062-1063. doi:10.3233/shti220268

12. Kannan V, Fish JS, Mutz JM, et al. Rapid Development of Specialty Population Registries and Quality Measures from Electronic Health Record Data*. An Agile Framework. *Methods Inf Med*. Jun 14 2017;56(99):e74-e83. doi:10.3414/me16-02-0031

13. Kapoor R, Sleeman WCt, Nalluri JJ, et al. Automated data abstraction for quality surveillance and outcome assessment in radiation oncology. *J Appl Clin Med Phys*. Jul 2021;22(7):177-187. doi:10.1002/acm2.13308

14. Kariuki JM, Manders EJ, Richards J, et al. Automating indicator data reporting from health facility EMR to a national aggregate data system in Kenya: An Interoperability field-test using OpenMRS and DHIS2. *Online J Public Health Inform*. 2016;8(2):e188. doi:10.5210/ojphi.v8i2.6722

15. Li N, Zhu Q, Dang Y, et al. Development and Implementation of a Dynamically Updated Big Data Intelligence Platform Using Electronic Medical Records for Secondary Hypertension. *Rev Cardiovasc Med*. Mar 2024;25(3):104. doi:10.31083/j.rcm2503104

16. Milinovich A, Kattan MW. Extracting and utilizing electronic health data from Epic for research. *Ann Transl Med*. Feb 2018;6(3):42. doi:10.21037/atm.2018.01.13

17. Miyake M, Akiyama M, Kashiwagi K, Sakamoto T, Oshika T. Japan Ocular Imaging Registry: a national ophthalmology real-world database. *Jpn J Ophthalmol*. Nov 2022;66(6):499-503. doi:10.1007/s10384-022-00941-0

18. Mou Z, Sitapati AM, Ramachandran M, Doucet JJ, Liepert AE. Development and implementation of an automated electronic health record-linked registry for emergency general surgery. *J Trauma Acute Care Surg*. Aug 1 2022;93(2):273-279. doi:10.1097/ta.0000000000003582

19. Mou Y, Lehmkuhl J, Sauerbrunn N, et al. Improving the Quality of Unstructured Cancer Data Using Large Language Models: A German Oncological Case Study. *Stud Health Technol Inform*. Aug 22 2024;316:685-689. doi:10.3233/shti240507

20. Munzone E, Marra A, Comotto F, et al. Development and Validation of a Natural Language Processing Algorithm for Extracting Clinical and Pathological Features of Breast Cancer From Pathology Reports. *JCO Clin Cancer Inform*. Aug 2024;8:e2400034. doi:10.1200/cci.24.00034

21. Nakagawa N, Sofue T, Kanda E, et al. J-CKD-DB: a nationwide multicentre electronic health record-based chronic kidney disease database in Japan. *Sci Rep*. Apr 30 2020;10(1):7351. doi:10.1038/s41598-020-64123-z

22. Nasir K, Gullapelli R, Nicolas JC, et al. Houston Methodist cardiovascular learning health system (CVD-LHS) registry: Methods for development and implementation of an automated electronic medical record-based registry using an informatics framework approach. *Am J Prev Cardiol*. Jun 2024;18:100678. doi:10.1016/j.ajpc.2024.100678

23. Nathan JK, Foley J, Hoang T, et al. The stroke navigator: meaningful use of the electronic health record to efficiently report inpatient stroke care quality. *J Am Med Inform Assoc*. Nov 1 2018;25(11):1534-1539. doi:10.1093/jamia/ocy102

24. Pan HY, Shaitelman SF, Perkins GH, Schlembach PJ, Woodward WA, Smith BD. Implementing a Real-Time Electronic Data Capture System to Improve Clinical Documentation in Radiation Oncology. *J Am Coll Radiol*. Apr 2016;13(4):401-7. doi:10.1016/j.jacr.2015.09.036

25. Pittman CA, Miranpuri AS. Neurosurgery clinical registry data collection utilizing Informatics for Integrating Biology and the Bedside and electronic health records at the University of Rochester. *Neurosurg Focus*. Dec 2015;39(6):E16. doi:10.3171/2015.9.Focus15382

26. Rayman S, Benvenisti H, Westrich G, Schtrechman G, Nissan A, Segev L. Colorectal Surgery Surveillance: A Novel Method for Composing an Automated Real-time Prospective Registry. *Isr Med Assoc J*. Apr 2021;23(4):239-244.

27. Rubio-Mayo P, Ojeda-Thies C, Jiménez-Cerezo MJ, Garcia-Barrio N, Cruz-Bermúdez JL, Pedrera-Jiménez M. HCE2RNFC: An Efficient Methodology for Reusing the EHR in the Spanish National Hip Fracture Registry. *Stud Health Technol Inform*. Aug 22 2024;316:1422-1426. doi:10.3233/shti240679

28. Salati M, Pompili C, Refai M, Xiumè F, Sabbatini A, Brunelli A. Real-time database drawn from an electronic health record for a thoracic surgery unit: high-quality clinical data saving time and human resources†. *European Journal of Cardio-Thoracic Surgery*. 2014;45(6):1017-1019. doi:10.1093/ejcts/ezt577

29. Shalhout SZ, Saqlain F, Wright K, Akinyemi O, Miller DM. Generalizable EHR-R-REDCap pipeline for a national multi-institutional rare tumor patient registry. *JAMIA Open*. Apr 2022;5(1):ooab118. doi:10.1093/jamiaopen/ooab118

30. Stevens A, Karki S, Shivers E, et al. SmartChart Suite: a Fast Healthcare Interoperability Resources-based framework for longitudinal syphilis surveillance using structured and unstructured data. *JAMIA Open*. Feb 2025;8(1):ooae145. doi:10.1093/jamiaopen/ooae145

31. Sugiyama T, Miyo K, Tsujimoto T, et al. Design of and rationale for the Japan Diabetes compREhensive database project based on an Advanced electronic Medical record System (J-DREAMS). *Diabetol Int*. Nov 2017;8(4):375-382. doi:10.1007/s13340-017-0326-y

32. Tavabi N, Pruneski J, Golchin S, et al. Building large-scale registries from unstructured clinical notes using a low-resource natural language processing pipeline. *Artif Intell Med*. May 2024;151:102847. doi:10.1016/j.artmed.2024.102847

33. Valencia Morales DJ, Bansal V, Heavner SF, et al. Validation of automated data abstraction for SCCM discovery VIRUS COVID-19 registry: practical EHR export pathways (VIRUS-PEEP). *Front Med (Lausanne)*. 2023;10:1089087. doi:10.3389/fmed.2023.1089087

34. Wang B, Lai J, Cao H, et al. Enhancing the interoperability and transparency of real-world data extraction in clinical research: evaluating the feasibility and impact of a ChatGLM implementation in Chinese hospital settings. *Eur Heart J Digit Health*. Nov 2024;5(6):712-724. doi:10.1093/ehjdh/ztae066

35. Williams A, Goedicke W, Tissera KA, Mankarious LA. Leveraging Existing Tools in Electronic Health Record Systems to Automate Clinical Registry Compilation. *Otolaryngol Head Neck Surg*. Mar 2020;162(3):408-409. doi:10.1177/0194599820901713

36. Wulff A, Mast M, Hassler M, Montag S, Marschollek M, Jack T. Designing an openEHR-Based Pipeline for Extracting and Standardizing Unstructured Clinical Data Using Natural Language Processing. *Methods Inf Med*. Dec 2020;59(S 02):e64-e78. doi:10.1055/s-0040-1716403
