# Supplementary material for: Exploring Technological Solutions for Interoperability Between Patient Electronic Medical Records and Clinical Registries: Scoping Review
Source: J Med Internet Res. 2026 May 25;28:e82380. doi: 10.2196/82380 (PMC13200772; doi:10.2196/82380)
Supplement: Multimedia Appendix 6 [file jmir-v28-e82380-s006.docx]

**Table S1.** Summary of data privacy and security measures.

|  | **De-identification** | **Transfer Methodologies and Security** | | **Infrastructure and Storage Security** | | **Data Access Control and Authentication** | |
| --- | --- | --- | --- | --- | --- | --- | --- |
| **Author (year)** | ***Y/N (Method)*** | ***Y/N*** | ***Method utilized*** | ***Y/N*** | ***Security employed*** | ***Y/N*** | ***Methods for Data Access Control*** |
| Abu-Rish Blakeney et al.^1^ | Yes *(Not Reported)* | Yes | Not reported | Yes | HIPAA compliant housing | Yes | Not reported |
| Bacchi et al.^2^ | Not reported | No | Not reported | No | Not reported | No | Not reported |
| Bodagh et al.^3^ | Not reported | No | Not reported | No | Not reported | No | Not reported |
| Chen et al.^4^ | Yes *(Not Reported)* | No | Not reported | No | Not reported | No | Not reported |
| Cheng et al.^5^ | Not reported | Yes | Not Reported | Yes | HIPAA compliant storage | Yes | Token-based access control, RBAC, audit logs |
| Dalhatu et al.^6^ | Yes *(Not Reported)* | Yes | HTTPS | Yes | Secure web-based interface | Yes | RBAC, audit logs |
| Dong et al.^7^ | Yes *(Not Reported)* | No | Not reported | Yes | Intranet access only | Yes | Only external access restricted with password and USB key |
| Garies et al.^8^ | Yes *(Algorithms)* | Yes | SFTP | Yes | Centralized secure hosting | Yes | Audit logs, external access restricted with password and USB key |
| Goel et al.^9^ | Yes *(Not Reported)* | Yes | HTTPS | No | Not reported | No | Not reported |
| González et al.^10^ | Yes *(Not Reported)* | No | Not reported | No | Not reported | No | Not reported |
| Heider et al.^11^ | Not reported | Yes | Custom SSL | No | Not reported | No | Not reported |
| Kannan et al.^12^ | Not reported | No | Not reported | No | Not reported | No | Not reported |
| Kapoor et al.^13^ | Yes *(Manual)* | Yes | Not Reported | Yes | HIPAA compliant storage | Yes | SSO and MFA |
| Kariuki et al.^14^ | Not reported | No | Not reported | No | Not reported | No | Not reported |
| Li et al.^15^ | Yes *(Not Reported)* | No | Not reported | No | Not reported | Yes | Permission set access control |
| Milinovich et al.^16^ | Not reported | No | Not reported | No | Not reported | No | Not reported |
| Miyake et al.^17^ | Yes *(Not Reported)* | Yes | SIN and VPN | Yes | Secure cloud infrastructure | Yes | Not reported |
| Mou et al.^18^ | Not reported | No | Not reported | No | Not reported | Yes | Not reported |
| Mou et al.^19^ | Yes *(Not Reported)* | No | Not reported | No | Not reported | No | Not reported |
| Munzone et al.^20^ | Yes *(Not Reported)* | No | Not reported | Yes | Secure cloud infrastructure | Yes | MFA |
| Nakagawa et al.^21^ | Yes *(Not Reported)* | Yes | HTTPS | Yes | Not Reported | Yes | Not Reported |
| Nasir et al.^22^ | Yes *(Not Reported)* | No | Not reported | No | Not reported | Yes | Not Reported |
| Nathan et al.^23^ | Not reported | No | Not reported | No | Not reported | No | Not reported |
| Pan et al.^24^ | Not reported | Yes | SSL | No | Not reported | Yes | Audit logs |
| Pittman et al.^25^ | Yes *(Not Reported)* | No | Not reported | No | Not reported | Yes | RBAC |
| Rayman et al.^26^ | Not reported | No | Not reported | No | Not reported | Yes | Not Reported |
| Rubio-Mayo et al.^27^ | Not reported | No | Not reported | No | Not reported | No | Not reported |
| Salati et al.^28^ | Not reported | No | Not reported | No | Not reported | No | Not reported |
| Shalhout et al.^29^ | Yes *(Pipeline)* | No | Not reported | No | Not reported | No | Not reported |
| Stevens et al.^30^ | Not reported | No | Not reported | No | Not reported | Yes | Not reported |
| Sugiyama et al.^31^ | Yes *(Server)* | Yes | Various (e.g. VPN, LAN) | Yes | Centralized secure housing | Yes | Not Reported |
| Tavabi et al.^32^ | Not reported | No | Not reported | No | Not reported | No | Not reported |
| Valencia Morales et al.^33^ | Yes *(Not Reported)* | Yes | SFTP | No | Not reported | No | Not reported |
| Wang et al.^34^ | Yes *(Not Reported)* | No | Not reported | No | Not reported | No | Not reported |
| Williams et al.^35^ | Not reported | No | Not reported | No | Not reported | No | Not reported |
| Wulff et al.^36^ | Not reported | No | Not reported | No | Not reported | No | Not reported |

*(****Abbreviations****: HIPAA = Health Insurance Portability and Accountability Act, SFTP = Secure File Transfer Protocol, HTTPS = Hypertext Transfer Protocol Secure, RBAC = Role-Based Access Controls, SSL = Secure Sockets Layer, SSO = Single Sign On, MFA = Multi-Factor Authentication, SIN = Science Information Network, VPN = Virtual Private Network, LAN = Local Area Network, RBAC = Role-based access control)*

**REFERENCE LIST**

1. Abu-Rish Blakeney E, Wolpin S, Lavallee DC, Dardas T, Cheng R, Zierler B. Developing and implementing a heart failure data mart for research and quality improvement. *Inform Health Soc Care*. 2019;44(2):164-175. doi:10.1080/17538157.2018.1455202

2. Bacchi S, Gluck S, Koblar S, Jannes J, Kleinig T. Automated information extraction from free-text medical documents for stroke key performance indicators: a pilot study. *Intern Med J*. Feb 2022;52(2):315-317. doi:10.1111/imj.15678

3. Bodagh N, Archbold RA, Weerackody R, et al. Feasibility of real-time capture of routine clinical data in the electronic health record: a hospital-based, observational service-evaluation study. *BMJ Open*. 2018;8(3):e019790. doi:10.1136/bmjopen-2017-019790

4. Chen AM, Kupelian PA, Wang PC, Steinberg ML. Development of a Radiation Oncology-Specific Prospective Data Registry for Research and Quality Improvement: A Clinical Workflow-Based Solution. *JCO Clin Cancer Inform*. Dec 2018;2:1-9. doi:10.1200/cci.17.00036

5. Cheng AC, Duda SN, Taylor R, et al. REDCap on FHIR: Clinical Data Interoperability Services. *Journal of Biomedical Informatics*. 2021/09/01/ 2021;121:103871. doi:<https://doi.org/10.1016/j.jbi.2021.103871>

6. Dalhatu I, Aniekwe C, Bashorun A, et al. From Paper Files to Web-Based Application for Data-Driven Monitoring of HIV Programs: Nigeria's Journey to a National Data Repository for Decision-Making and Patient Care. *Methods Inf Med*. Sep 2023;62(3-04):130-139. doi:10.1055/s-0043-1768711

7. Dong Y, Fang K, Wang X, et al. The network of Shanghai Stroke Service System (4S): A public health-care web-based database using automatic extraction of electronic medical records. *Int J Stroke*. Jul 2018;13(5):539-544. doi:10.1177/1747493018765492

8. Garies S, Cummings M, Forst B, et al. Achieving quality primary care data: a description of the Canadian Primary Care Sentinel Surveillance Network data capture, extraction, and processing in Alberta. *Int J Popul Data Sci*. Jul 29 2019;4(2):1132. doi:10.23889/ijpds.v4i2.1132

9. Goel AK, Campbell WS, Moldwin R. Structured Data Capture for Oncology. *JCO Clin Cancer Inform*. Feb 2021;5:194-201. doi:10.1200/cci.20.00103

10. González L, Pérez-Rey D, Alonso E, et al. Building an i2b2-Based Population Repository for Clinical Research. *Stud Health Technol Inform*. Jun 16 2020;270:78-82. doi:10.3233/shti200126

11. Heider PM, Pipaliya RM, Meystre SM. A Natural Language Processing Tool Offering Data Extraction for COVID-19 Related Information (DECOVRI). *Stud Health Technol Inform*. Jun 6 2022;290:1062-1063. doi:10.3233/shti220268

12. Kannan V, Fish JS, Mutz JM, et al. Rapid Development of Specialty Population Registries and Quality Measures from Electronic Health Record Data*. An Agile Framework. *Methods Inf Med*. Jun 14 2017;56(99):e74-e83. doi:10.3414/me16-02-0031

13. Kapoor R, Sleeman WCt, Nalluri JJ, et al. Automated data abstraction for quality surveillance and outcome assessment in radiation oncology. *J Appl Clin Med Phys*. Jul 2021;22(7):177-187. doi:10.1002/acm2.13308

14. Kariuki JM, Manders EJ, Richards J, et al. Automating indicator data reporting from health facility EMR to a national aggregate data system in Kenya: An Interoperability field-test using OpenMRS and DHIS2. *Online J Public Health Inform*. 2016;8(2):e188. doi:10.5210/ojphi.v8i2.6722

15. Li N, Zhu Q, Dang Y, et al. Development and Implementation of a Dynamically Updated Big Data Intelligence Platform Using Electronic Medical Records for Secondary Hypertension. *Rev Cardiovasc Med*. Mar 2024;25(3):104. doi:10.31083/j.rcm2503104

16. Milinovich A, Kattan MW. Extracting and utilizing electronic health data from Epic for research. *Ann Transl Med*. Feb 2018;6(3):42. doi:10.21037/atm.2018.01.13

17. Miyake M, Akiyama M, Kashiwagi K, Sakamoto T, Oshika T. Japan Ocular Imaging Registry: a national ophthalmology real-world database. *Jpn J Ophthalmol*. Nov 2022;66(6):499-503. doi:10.1007/s10384-022-00941-0

18. Mou Z, Sitapati AM, Ramachandran M, Doucet JJ, Liepert AE. Development and implementation of an automated electronic health record-linked registry for emergency general surgery. *J Trauma Acute Care Surg*. Aug 1 2022;93(2):273-279. doi:10.1097/ta.0000000000003582

19. Mou Y, Lehmkuhl J, Sauerbrunn N, et al. Improving the Quality of Unstructured Cancer Data Using Large Language Models: A German Oncological Case Study. *Stud Health Technol Inform*. Aug 22 2024;316:685-689. doi:10.3233/shti240507

20. Munzone E, Marra A, Comotto F, et al. Development and Validation of a Natural Language Processing Algorithm for Extracting Clinical and Pathological Features of Breast Cancer From Pathology Reports. *JCO Clin Cancer Inform*. Aug 2024;8:e2400034. doi:10.1200/cci.24.00034

21. Nakagawa N, Sofue T, Kanda E, et al. J-CKD-DB: a nationwide multicentre electronic health record-based chronic kidney disease database in Japan. *Sci Rep*. Apr 30 2020;10(1):7351. doi:10.1038/s41598-020-64123-z

22. Nasir K, Gullapelli R, Nicolas JC, et al. Houston Methodist cardiovascular learning health system (CVD-LHS) registry: Methods for development and implementation of an automated electronic medical record-based registry using an informatics framework approach. *Am J Prev Cardiol*. Jun 2024;18:100678. doi:10.1016/j.ajpc.2024.100678

23. Nathan JK, Foley J, Hoang T, et al. The stroke navigator: meaningful use of the electronic health record to efficiently report inpatient stroke care quality. *J Am Med Inform Assoc*. Nov 1 2018;25(11):1534-1539. doi:10.1093/jamia/ocy102

24. Pan HY, Shaitelman SF, Perkins GH, Schlembach PJ, Woodward WA, Smith BD. Implementing a Real-Time Electronic Data Capture System to Improve Clinical Documentation in Radiation Oncology. *J Am Coll Radiol*. Apr 2016;13(4):401-7. doi:10.1016/j.jacr.2015.09.036

25. Pittman CA, Miranpuri AS. Neurosurgery clinical registry data collection utilizing Informatics for Integrating Biology and the Bedside and electronic health records at the University of Rochester. *Neurosurg Focus*. Dec 2015;39(6):E16. doi:10.3171/2015.9.Focus15382

26. Rayman S, Benvenisti H, Westrich G, Schtrechman G, Nissan A, Segev L. Colorectal Surgery Surveillance: A Novel Method for Composing an Automated Real-time Prospective Registry. *Isr Med Assoc J*. Apr 2021;23(4):239-244.

27. Rubio-Mayo P, Ojeda-Thies C, Jiménez-Cerezo MJ, Garcia-Barrio N, Cruz-Bermúdez JL, Pedrera-Jiménez M. HCE2RNFC: An Efficient Methodology for Reusing the EHR in the Spanish National Hip Fracture Registry. *Stud Health Technol Inform*. Aug 22 2024;316:1422-1426. doi:10.3233/shti240679

28. Salati M, Pompili C, Refai M, Xiumè F, Sabbatini A, Brunelli A. Real-time database drawn from an electronic health record for a thoracic surgery unit: high-quality clinical data saving time and human resources†. *European Journal of Cardio-Thoracic Surgery*. 2014;45(6):1017-1019. doi:10.1093/ejcts/ezt577

29. Shalhout SZ, Saqlain F, Wright K, Akinyemi O, Miller DM. Generalizable EHR-R-REDCap pipeline for a national multi-institutional rare tumor patient registry. *JAMIA Open*. Apr 2022;5(1):ooab118. doi:10.1093/jamiaopen/ooab118

30. Stevens A, Karki S, Shivers E, et al. SmartChart Suite: a Fast Healthcare Interoperability Resources-based framework for longitudinal syphilis surveillance using structured and unstructured data. *JAMIA Open*. Feb 2025;8(1):ooae145. doi:10.1093/jamiaopen/ooae145

31. Sugiyama T, Miyo K, Tsujimoto T, et al. Design of and rationale for the Japan Diabetes compREhensive database project based on an Advanced electronic Medical record System (J-DREAMS). *Diabetol Int*. Nov 2017;8(4):375-382. doi:10.1007/s13340-017-0326-y

32. Tavabi N, Pruneski J, Golchin S, et al. Building large-scale registries from unstructured clinical notes using a low-resource natural language processing pipeline. *Artif Intell Med*. May 2024;151:102847. doi:10.1016/j.artmed.2024.102847

33. Valencia Morales DJ, Bansal V, Heavner SF, et al. Validation of automated data abstraction for SCCM discovery VIRUS COVID-19 registry: practical EHR export pathways (VIRUS-PEEP). *Front Med (Lausanne)*. 2023;10:1089087. doi:10.3389/fmed.2023.1089087

34. Wang B, Lai J, Cao H, et al. Enhancing the interoperability and transparency of real-world data extraction in clinical research: evaluating the feasibility and impact of a ChatGLM implementation in Chinese hospital settings. *Eur Heart J Digit Health*. Nov 2024;5(6):712-724. doi:10.1093/ehjdh/ztae066

35. Williams A, Goedicke W, Tissera KA, Mankarious LA. Leveraging Existing Tools in Electronic Health Record Systems to Automate Clinical Registry Compilation. *Otolaryngol Head Neck Surg*. Mar 2020;162(3):408-409. doi:10.1177/0194599820901713

36. Wulff A, Mast M, Hassler M, Montag S, Marschollek M, Jack T. Designing an openEHR-Based Pipeline for Extracting and Standardizing Unstructured Clinical Data Using Natural Language Processing. *Methods Inf Med*. Dec 2020;59(S 02):e64-e78. doi:10.1055/s-0040-1716403
