# Supplementary material for: Exploring Technological Solutions for Interoperability Between Patient Electronic Medical Records and Clinical Registries: Scoping Review
Source: J Med Internet Res. 2026 May 25;28:e82380. doi: 10.2196/82380 (PMC13200772; doi:10.2196/82380)
Supplement: Multimedia Appendix 8 [file jmir-v28-e82380-s008.docx]

## **Table S1.** Summary of implementation challenges identified in establishing EMR-to-registry interoperability.

| **Category** | **Description and Details** |
| --- | --- |
| Data Quality | Missing data or incomplete documentation^1-6^  Incomplete data due to annotation limitations^7^  Incomplete data capture limited by data format^8-10^  Inaccurate data input or extraction^7,11-15^  Inaccurate data due to retention of pre-populated data^16^  Data quality concerns due to interpretive discrepancies^10^ |
| Data Mapping, Standardization and Semantic Harmonization | Locating and defining variables ^17^  Mapping complexity due to variable documentation locations^8^  Lack of a standardized data dictionary^4^  Lack of documentation standardization^1,10,18^  Lack of established standards for primary care^19^  Inconsistent availability and implementation of FHIR across vendors and institutions^20^  Need for custom mapping due to inconsistent alignment with reference coding systems^21,22^  Semantic challenge in concept mapping due to overlapping clinical terms^23^  Lack of semantic harmonization and standardization across institutions^10,22,24^ |
| Technical, Infrastructure and Resource Availability | Time consuming exchange and transfer^23,25^  Software, system and technical complexity^5,19^  Logistical and infrastructure setup hurdles^26,27^  Other technical challenges not specified^20^  Significant time and resource investment required for system development and implementation^9,10,14,18,21,28^  Dependence on network performance for timeliness of data^9^  Technical and clinical informatics expertise availability and dependence ^11,27^  Vendor and institutional constraints, capacity and limitations^10,11^ |
| Workflow Integration and Adoption | Poor staff buy-in, uptake or adoption^6,16,20,29,30^  Perceived time burden^29^  Patient enrolment burden^30^  Burden on clinical workflow^6^  Clinical burden from non-clinical documentation requirements^14,16^  Preference for unstructured input due to limitations in narrative expressivity in SDC^16^  Variability in documentation and workflows across institutions^6,11^ |
| Interoperability and Data Integrations Across Systems | Inability to capture events outside the institution^2,4,5,7,17^  Collaboration across multiple networks^19^  Extracting and aggregating data from disparate sources^31^  Readiness gaps in data standardization and cross-institutional interoperability^32^ |
| Privacy, Security and Governance | Policy and governance challenges^20^  Ethics committee negotiations and procedural hurdles^26^  Privacy concerns related to use of artificial intelligence in clinical contexts^12^ |
| Registry Scope, Generalizability and Maintenance | Defining registry scope due to competing stakeholder and research priorities^11,17^  Balancing data volume with relevance in registry capture^20^  Ongoing maintenance and cost^11,33^  Limited generalizability due to limited scope ^12,24,33^ |
| Semantic and Temporal Issues | Inability to identify patients in real-time^17^  Inability to incorporate retrospective temporal information^15^ |

*(N.B. SDC = Structured data capture)*

**REFERENCE LIST**

1. Bacchi S, Gluck S, Koblar S, Jannes J, Kleinig T. Automated information extraction from free-text medical documents for stroke key performance indicators: a pilot study. *Intern Med J*. Feb 2022;52(2):315-317. doi:10.1111/imj.15678

2. Chen AM, Kupelian PA, Wang PC, Steinberg ML. Development of a Radiation Oncology-Specific Prospective Data Registry for Research and Quality Improvement: A Clinical Workflow-Based Solution. *JCO Clin Cancer Inform*. Dec 2018;2:1-9. doi:10.1200/cci.17.00036

3. Kariuki JM, Manders EJ, Richards J, et al. Automating indicator data reporting from health facility EMR to a national aggregate data system in Kenya: An Interoperability field-test using OpenMRS and DHIS2. *Online J Public Health Inform*. 2016;8(2):e188. doi:10.5210/ojphi.v8i2.6722

4. Li N, Zhu Q, Dang Y, et al. Development and Implementation of a Dynamically Updated Big Data Intelligence Platform Using Electronic Medical Records for Secondary Hypertension. *Rev Cardiovasc Med*. Mar 2024;25(3):104. doi:10.31083/j.rcm2503104

5. Nasir K, Gullapelli R, Nicolas JC, et al. Houston Methodist cardiovascular learning health system (CVD-LHS) registry: Methods for development and implementation of an automated electronic medical record-based registry using an informatics framework approach. *Am J Prev Cardiol*. Jun 2024;18:100678. doi:10.1016/j.ajpc.2024.100678

6. Rayman S, Benvenisti H, Westrich G, Schtrechman G, Nissan A, Segev L. Colorectal Surgery Surveillance: A Novel Method for Composing an Automated Real-time Prospective Registry. *Isr Med Assoc J*. Apr 2021;23(4):239-244.

7. Munzone E, Marra A, Comotto F, et al. Development and Validation of a Natural Language Processing Algorithm for Extracting Clinical and Pathological Features of Breast Cancer From Pathology Reports. *JCO Clin Cancer Inform*. Aug 2024;8:e2400034. doi:10.1200/cci.24.00034

8. Pittman CA, Miranpuri AS. Neurosurgery clinical registry data collection utilizing Informatics for Integrating Biology and the Bedside and electronic health records at the University of Rochester. *Neurosurg Focus*. Dec 2015;39(6):E16. doi:10.3171/2015.9.Focus15382

9. Shalhout SZ, Saqlain F, Wright K, Akinyemi O, Miller DM. Generalizable EHR-R-REDCap pipeline for a national multi-institutional rare tumor patient registry. *JAMIA Open*. Apr 2022;5(1):ooab118. doi:10.1093/jamiaopen/ooab118

10. Valencia Morales DJ, Bansal V, Heavner SF, et al. Validation of automated data abstraction for SCCM discovery VIRUS COVID-19 registry: practical EHR export pathways (VIRUS-PEEP). *Front Med (Lausanne)*. 2023;10:1089087. doi:10.3389/fmed.2023.1089087

11. Mou Z, Sitapati AM, Ramachandran M, Doucet JJ, Liepert AE. Development and implementation of an automated electronic health record-linked registry for emergency general surgery. *J Trauma Acute Care Surg*. Aug 1 2022;93(2):273-279. doi:10.1097/ta.0000000000003582

12. Mou Y, Lehmkuhl J, Sauerbrunn N, et al. Improving the Quality of Unstructured Cancer Data Using Large Language Models: A German Oncological Case Study. *Stud Health Technol Inform*. Aug 22 2024;316:685-689. doi:10.3233/shti240507

13. Salati M, Pompili C, Refai M, Xiumè F, Sabbatini A, Brunelli A. Real-time database drawn from an electronic health record for a thoracic surgery unit: high-quality clinical data saving time and human resources†. *European Journal of Cardio-Thoracic Surgery*. 2014;45(6):1017-1019. doi:10.1093/ejcts/ezt577

14. Wang B, Lai J, Cao H, et al. Enhancing the interoperability and transparency of real-world data extraction in clinical research: evaluating the feasibility and impact of a ChatGLM implementation in Chinese hospital settings. *Eur Heart J Digit Health*. Nov 2024;5(6):712-724. doi:10.1093/ehjdh/ztae066

15. Wulff A, Mast M, Hassler M, Montag S, Marschollek M, Jack T. Designing an openEHR-Based Pipeline for Extracting and Standardizing Unstructured Clinical Data Using Natural Language Processing. *Methods Inf Med*. Dec 2020;59(S 02):e64-e78. doi:10.1055/s-0040-1716403

16. Nathan JK, Foley J, Hoang T, et al. The stroke navigator: meaningful use of the electronic health record to efficiently report inpatient stroke care quality. *J Am Med Inform Assoc*. Nov 1 2018;25(11):1534-1539. doi:10.1093/jamia/ocy102

17. Abu-Rish Blakeney E, Wolpin S, Lavallee DC, Dardas T, Cheng R, Zierler B. Developing and implementing a heart failure data mart for research and quality improvement. *Inform Health Soc Care*. 2019;44(2):164-175. doi:10.1080/17538157.2018.1455202

18. Tavabi N, Pruneski J, Golchin S, et al. Building large-scale registries from unstructured clinical notes using a low-resource natural language processing pipeline. *Artif Intell Med*. May 2024;151:102847. doi:10.1016/j.artmed.2024.102847

19. Garies S, Cummings M, Forst B, et al. Achieving quality primary care data: a description of the Canadian Primary Care Sentinel Surveillance Network data capture, extraction, and processing in Alberta. *Int J Popul Data Sci*. Jul 29 2019;4(2):1132. doi:10.23889/ijpds.v4i2.1132

20. Cheng AC, Duda SN, Taylor R, et al. REDCap on FHIR: Clinical Data Interoperability Services. *Journal of Biomedical Informatics*. 2021/09/01/ 2021;121:103871. doi:<https://doi.org/10.1016/j.jbi.2021.103871>

21. González L, Pérez-Rey D, Alonso E, et al. Building an i2b2-Based Population Repository for Clinical Research. *Stud Health Technol Inform*. Jun 16 2020;270:78-82. doi:10.3233/shti200126

22. Nakagawa N, Sofue T, Kanda E, et al. J-CKD-DB: a nationwide multicentre electronic health record-based chronic kidney disease database in Japan. *Sci Rep*. Apr 30 2020;10(1):7351. doi:10.1038/s41598-020-64123-z

23. Heider PM, Pipaliya RM, Meystre SM. A Natural Language Processing Tool Offering Data Extraction for COVID-19 Related Information (DECOVRI). *Stud Health Technol Inform*. Jun 6 2022;290:1062-1063. doi:10.3233/shti220268

24. Sugiyama T, Miyo K, Tsujimoto T, et al. Design of and rationale for the Japan Diabetes compREhensive database project based on an Advanced electronic Medical record System (J-DREAMS). *Diabetol Int*. Nov 2017;8(4):375-382. doi:10.1007/s13340-017-0326-y

25. Dalhatu I, Aniekwe C, Bashorun A, et al. From Paper Files to Web-Based Application for Data-Driven Monitoring of HIV Programs: Nigeria's Journey to a National Data Repository for Decision-Making and Patient Care. *Methods Inf Med*. Sep 2023;62(3-04):130-139. doi:10.1055/s-0043-1768711

26. Miyake M, Akiyama M, Kashiwagi K, Sakamoto T, Oshika T. Japan Ocular Imaging Registry: a national ophthalmology real-world database. *Jpn J Ophthalmol*. Nov 2022;66(6):499-503. doi:10.1007/s10384-022-00941-0

27. Stevens A, Karki S, Shivers E, et al. SmartChart Suite: a Fast Healthcare Interoperability Resources-based framework for longitudinal syphilis surveillance using structured and unstructured data. *JAMIA Open*. Feb 2025;8(1):ooae145. doi:10.1093/jamiaopen/ooae145

28. Williams A, Goedicke W, Tissera KA, Mankarious LA. Leveraging Existing Tools in Electronic Health Record Systems to Automate Clinical Registry Compilation. *Otolaryngol Head Neck Surg*. Mar 2020;162(3):408-409. doi:10.1177/0194599820901713

29. Bodagh N, Archbold RA, Weerackody R, et al. Feasibility of real-time capture of routine clinical data in the electronic health record: a hospital-based, observational service-evaluation study. *BMJ Open*. 2018;8(3):e019790. doi:10.1136/bmjopen-2017-019790

30. Kannan V, Fish JS, Mutz JM, et al. Rapid Development of Specialty Population Registries and Quality Measures from Electronic Health Record Data*. An Agile Framework. *Methods Inf Med*. Jun 14 2017;56(99):e74-e83. doi:10.3414/me16-02-0031

31. Kapoor R, Sleeman WCt, Nalluri JJ, et al. Automated data abstraction for quality surveillance and outcome assessment in radiation oncology. *J Appl Clin Med Phys*. Jul 2021;22(7):177-187. doi:10.1002/acm2.13308

32. Rubio-Mayo P, Ojeda-Thies C, Jiménez-Cerezo MJ, Garcia-Barrio N, Cruz-Bermúdez JL, Pedrera-Jiménez M. HCE2RNFC: An Efficient Methodology for Reusing the EHR in the Spanish National Hip Fracture Registry. *Stud Health Technol Inform*. Aug 22 2024;316:1422-1426. doi:10.3233/shti240679

33. Pan HY, Shaitelman SF, Perkins GH, Schlembach PJ, Woodward WA, Smith BD. Implementing a Real-Time Electronic Data Capture System to Improve Clinical Documentation in Radiation Oncology. *J Am Coll Radiol*. Apr 2016;13(4):401-7. doi:10.1016/j.jacr.2015.09.036
